# An Exact Theory of Causal Emergence for Linear Stochastic Iteration Systems

**DOI:** 10.3390/e26080618

**Published:** 2024-07-23

**Authors:** Kaiwei Liu, Bing Yuan, Jiang Zhang

**Affiliations:** 1 School of Systems Science, Beijing Normal University, Beijing 100875, China; 202221250005@mail.bnu.edu.cn; 2 Swarma Research, Beijing 102300, China; yuanbing_cn@hotmail.com

**Keywords:** causal emergence, effective information, linear stochastic iteration system, coarse-graining

## Abstract

After coarse-graining a complex system, the dynamics of its macro-state may exhibit more pronounced causal effects than those of its micro-state. This phenomenon, known as causal emergence, is quantified by the indicator of effective information. However, two challenges confront this theory: the absence of well-developed frameworks in continuous stochastic dynamical systems and the reliance on coarse-graining methodologies. In this study, we introduce an exact theoretic framework for causal emergence within linear stochastic iteration systems featuring continuous state spaces and Gaussian noise. Building upon this foundation, we derive an analytical expression for effective information across general dynamics and identify optimal linear coarse-graining strategies that maximize the degree of causal emergence when the dimension averaged uncertainty eliminated by coarse-graining has an upper bound. Our investigation reveals that the maximal causal emergence and the optimal coarse-graining methods are primarily determined by the principal eigenvalues and eigenvectors of the dynamic system’s parameter matrix, with the latter not being unique. To validate our propositions, we apply our analytical models to three simplified physical systems, comparing the outcomes with numerical simulations, and consistently achieve congruent results.

## 1. Introduction

Many complex systems [1] in reality, such as cities [2,3,4], companies [5,6,7], bird flocks [8,9], perception systems [10,11,12], brains [13,14,15], cells [16,17], molecules, etc., all exhibit emergent behaviors or statistical laws on a macro-level that cannot be simply derived from micro-level properties or dynamics. However, how to quantitatively characterize emergence has only recently garnered widespread attention [18,19,20,21,22,23,24]. The theories of causal emergence attempt to capture the conception of emergence from the point of view of causality. Intuitively, causal emergence refers to the phenomenon in dynamical systems where stronger causal effects can be obtained on macro-states by coarse-graining the micro-states [25]. There are three different ways to quantify the idea of causal emergence, which are based on effective information [22,23], partial information decomposition [15,21,24,26,27], and dynamical independence [10,14,20], respectively. In this paper, we mainly focus on the first way.

Erik Hoel introduced the initial quantitative theory for causal emergence, founded on Effective Information (EI) [22,23,25]. Nevertheless, the original framework is limited to quantifying discrete Markov chains in both the time domain and state space [28,29], overlooking continuous state spaces. To extend the causal emergence theory in continuous spaces, P. Chvykov and E. Hoel put forth the theory of causal geometry [30], wherein they devised a method for calculating EI in functional mappings across continuous state spaces [6,31]. Nonetheless, this theory solely explores general functional mappings and neglects the multi-step dynamical evolution, rendering it inapplicable to dynamical systems in continuous state spaces. Furthermore, all of Hoel’s theories [22,23,25,30,32,33] necessitate a predefined coarse-graining strategy to discern instances of causal emergence. While this coarse-graining strategy can be derived by maximizing EI for macro-dynamics, solving the optimization problem for continuous variables proves challenging [6,34,35].

There exist various approaches to coarse-grain the micro-states of complex systems, including manually designed renormalization methods [36], traditional dimensionality reduction techniques [37,38,39,40,41], and machine-learning-based methods for coarse-graining or renormalization [6,34,42]. To automatically discover coarse-graining strategies that optimize causal emergence, J. Zhang and K. Liu introduced a machine-learning framework known as the Neural Information Squeezer (NIS) [43], employing reversible neural networks. This framework facilitates the automatic extraction of effective coarse-graining strategies and macro-state dynamics, enabling the direct identification of causal emergence from diverse time series data. Subsequently, the research team proposed the enhanced NIS+ framework [35], which integrates the optimization of coarse-graining through EI maximization into machine learning. NIS and NIS+ have successfully addressed the challenge of identifying causal emergence in data by optimizing coarse-graining techniques and macro-dynamics within continuous stochastic dynamical systems through EI maximization. Nonetheless, machine-learning-based methods [44,45,46] rely heavily on data adequacy and the level of neural network training. While they can provide numerical solutions, they lack a ground truth for assessing the quality of model training and the reliability of outcome indicators.

To overcome the limitations identified in previous studies, this paper will first derive analytical solutions for EI and causal emergence in linear stochastic iterative systems. It will then determine the explicit expression for maximal causal emergence and the corresponding optimal coarse-graining strategies, under the condition that the dimension-averaged uncertainty eliminated by coarse-graining is constrained by a specified threshold. Subsequent to this, numerical simulation experiments will be conducted on simplified physical systems to validate the theoretical findings. Additionally, we will explore challenges such as maximizing causal emergence under an alternative condition involving constructing the coarse-graining map through a combination of dimensionality reduction projection and rotation, showcasing the interplay between determinism and non-degeneracy, examining the connection between maximizing causal emergence and minimizing prediction errors in dynamics, and addressing nonlinear dynamics. The primary objective of this research is to address deficiencies in EI related to Transitional Probability Matrices (TPM) [22,25] and causal geometry within a toy model context [30]. Furthermore, it aims to lay a theoretical groundwork to enhance the identification of causal emergence through machine learning [35,43].

## 2. Basic Notions and Problems Formulation

### 2.1. Continuous Linear Stochastic Iteration System

A stochastic iteration system [47] is a sequence of random variables, $x_{t}$, at different times, *t*, which usually represent the dynamic evolution of a certain quantity, such as stock prices, temperature changes, traffic flows, etc. This paper mainly focuses on linear high-dimensional stochastic iteration systems [48] to reduce our calculation.

For variable $x_{t}\in R^{n}$ at time *t* in the stochastic iteration system, the evolution of $x_{t}$ follows the stochastic iterative equation

(1) $$\begin{array}{c} x_{t+1}=Ax_{t}+\varepsilon_{t}. \end{array}$$

We define matrix $A\in R^{n\times n}$ as a dynamical parameter matrix. In Equation (1), $\varepsilon_{t}∼N\left(0,\Sigma \right)$ represents Gaussian noises with zero means and the covariance matrix $\Sigma \in R^{n\times n}$. We specify the ranks of matrices rk(A)=rk(Σ)=n. rk(·) means the rank of a matrix. We can easily observe that the probability of $x_{t+1}$ falling in a region centered with $Ax_{t}$.

### 2.2. Coarse-Graining

In the realm of linear stochastic iterative systems, we confine coarse-graining strategies to linear maps. Therefore, a direct approach entails employing the relatively straightforward projection matrix coarse-graining method.

Generally, the coarse-graining strategy can be represented as $\phi \left(x_{t}\right):R^{n}\to R^{k},k<n$. It is designed to map micro-states to a lower-dimensional space and to convert micro-states into macro-states.

**Definition** **1.** *(Coarse-graining strategy): For variable $x_{t}$ at time t in the linear stochastic iteration system, we define linear mapping*

(2) $$\begin{array}{c} \phi \left(x_{t}\right)=Wx_{t}, \end{array}$$

*where $W\in R^{k\times n}$, rk(W)=k, to map $x_{t}$ in high-dimensional space $R^{n}$ to $y_{t}$ in low-dimensional space $R^{k}$, achieving coarse-graining of variables*

(3) $$\begin{array}{c} y_{t}=\phi \left(x_{t}\right)=Wx_{t} \end{array}$$

*in the stochastic iteration system.*

### 2.3. Macro Dynamical System

The coarse-graining strategy can map a set of micro-states to specific macro-states, and thus can naturally derive the expressions from micro-state parameter matrices *A* and Σ for dynamical models on macro-state space, which can be described by macro-state parameter matrices $A_{M}$ and $\Sigma_{M}$. Since $W\in R^{k\times n}$ in the linear coarse-graining strategy is irreversible, we need the Moore–Penrose generalized inverse matrix [49] $W^{†}$ in matrix theory [50] to derive $A_{M}$.

According to Equations (1) and (3), we can get that

(4) $$\begin{array}{c} \begin{array}{cc} y_{t+1} & =Wx_{t+1}=WAx_{t}+W\varepsilon_{t}. \end{array} \end{array}$$

Due to the fact that both the micro-dynamical model itself and the coarse-graining strategies are linear transformations, and based on the properties of linear algebra, there is also a set of equations for the macro-dynamics. Thus, we hope that there is a matrix $A_{M}$ satisfying the following equation:

(5) $$\begin{array}{c} \begin{array}{cc} y_{t+1} & =A_{M}y_{t}+\varepsilon_{M,t}. \end{array} \end{array}$$

According to Equations (4) and (5) and $y_{t}=Wx_{t}$, we can get that

(6) $$\begin{array}{c} \left\{\begin{array}{c} WA=A_{M}W\\ W\varepsilon_{t}=\varepsilon_{M,t} \end{array}\right.. \end{array}$$

Since $\varepsilon_{t}∼N\left(0,\Sigma \right)$, $\varepsilon_{M,t}=W\varepsilon_{t}∼N\left(0,\Sigma_{M}\right)$ and $W\in R^{k\times n}$ is irreversible, we require the Moore–Penrose generalized inverse matrix $W^{†}\in R^{n\times k},WW^{†}=I_{k}$ to solve Equation (6). By multiplying both sides of the first equation of Equation (6) by $W^{†}$, we can obtain the macro-state parameter matrix,

(7) $$A_{M}=WAW^{†},$$

and the macro dynamics in the Definition 2 as follows.

**Definition** **2.** *(Macro dynamics): For the linear stochastic iteration system in Equation (1), the variables $x_{t}\in R^{n},t=0,1,\ldots$, are mapped to macro state variables $y_{t}\in R^{k},t=0,1,\ldots$, and there exists a new evolutionary dynamics between macro state variables, which we call macro dynamics. Macro dynamics can be represented by macro iterative equations,*

(8) $$\begin{array}{c} y_{t+1}=A_{M}y_{t}+\varepsilon_{M,t}, \end{array}$$

*in which $A_{M}=WAW^{†}\in R^{k\times k}$, $\varepsilon_{M,t}∼N\left(0,\Sigma_{M}\right),\Sigma_{M}=W\Sigma W^{T}$. $W^{†}$ is the Moore–Penrose generalized inverse matrix of W.*

### 2.4. Inverse-Coarse-Graining

To evaluate the information loss caused by coarse-graining and derive macro dynamics, we also need to transform the macro-state back to the micro-state, which is called inverse-coarse-graining. Due to the difference in dimensions *n* and *k* of the vectors before and after transformation, coarse-graining is not a reversible process. For linear transformations, irreversibility is manifested as $W\in R^{k\times n}$ in linear coarse-graining.

**Definition** **3.** *(Inverse-coarse-graining): As $W^{†}$ is the Moore–Penrose generalized inverse matrix of W, we define*

(9) $$\begin{array}{c} \phi^{†}\left(y_{t}\right)=W^{†}y_{t}, \end{array}$$

*$W^{†}\in R^{n\times k}$, $\mathrm{rk}(W^{†})=k$ as the inverse-coarse-graining mapping of variables in the stochastic iteration system. By applying the inverse-coarse-graining*

(10) $$\begin{array}{c} {\hat{x}}_{t}=W^{†}y_{t}, \end{array}$$

*we can map the variable $y_{t}\in R^{k}$ after dimensionality reduction back to a high-dimensional space, $R^{n}$.*

With inverse-coarse-graining, we can then estimate the prediction loss function of the dynamics after coarse-graining.

## 3. Effective Information and Causal Emergence

With the definitions and expressions of micro dynamics and macro dynamics, we can study the phenomenon of causal emergence. We start with the calculation of effective information and then use the method of difference of effective information between micro-dynamics and macro-dynamics to calculate the degree of causal emergence.

### 3.1. Effective Information

We refer to Hoel’s Effective Information (EI) metric to measure the magnitude of causal effects in dynamical systems. In general, EI can be understood as the mutual information between the probability distributions of the states of variables at two different time points when the state at the earlier time is intervened artificially and keeps the causal mechanism, i.e., the transitional probability $Pr(x_{t+1}|x_{t})$, unchanged, which is

(11) $$EI=I({\tilde{x}}_{t+1},x_{t}|do\left(x_{t}∼U\left(X\right)\right)),$$

where $x_{t}\in X$, and U(X) means the uniform distribution or maximum entropy distribution on *X*. The intervention is formalized using Judea Pearl’s theory [51] of causality, particularly through do(·) operations, which means artificially defining the probability distribution space of a variable. Consequently, the distribution of $x_{t+1}$ will be indirectly altered by the intervention on $x_{t}$ through the causal mechanism $Pr(x_{t+1}|x_{t})$. Therefore, ${\tilde{x}}_{t+1}$ denotes the random variable representing $x_{t+1}$ following the intervention on $x_{t}$. It should be noted that Equation (11) is only an operational definition and the intervention of the do(·) operator is just an imaginary operation to calculate EI but has no real physical meaning.

The most classic applications of EI are in the analysis of Transitional Probability Matrices (TPM) for Markov chains. Assuming our probability transition matrix is $M={\left(M_{ij}\right)}_{n\times n}={\left(M_{1}^{T},\ldots ,M_{i}^{T},\ldots ,M_{n}^{T}\right)}^{T}\in R^{n\times n}$, $M_{i}=\left(M_{i1},\ldots ,M_{ij},\ldots ,M_{in}\right)\in R^{1\times n}$, 1≤i, j≤n, for the intervention on the Markov transition matrix at *t*, let $P(X_{t}=x_{t})=1/n$, then the state at t+1 will follow the effect distribution $E_{D}=(\sum_{i}M_{i1}/n,\ldots ,\sum_{i}M_{ij}/n,\ldots ,$$\sum_{i}M_{in}/n)\in R^{1\times n}$. So, EI is [18]:

(12) $$\begin{array}{c} EI\left(M\right)=\frac{1}{n}\sum_{i=1}^{n}D_{KL}(M_{i}\left|\right|E_{D})=\frac{1}{n}\sum_{i,j}M_{ij}\log_{2}\left(\frac{nM_{ij}}{\sum_{l}M_{lj}}\right), \end{array}$$

where $D_{KL}\left(\cdot ||\cdot \right)$ is the KL-divergence between two probability distributions. The benefit of expressing Equation (11) to the new form of Equation (12) is to stress that EI is only the function of the dynamics (*M*) and independent of the distribution of $x_{t}$. This calculation method requires both variable time and state space to be discrete.

According to previous works [22,25,29], effective information, EI(M), can be divided into two parts, determinism $-{\langle H(M_{i}))\rangle }_{i}$ and non-degeneracy $H(E_{D})$, as

(13) $$\begin{array}{c} \begin{array}{cc} EI(M) & =-{\langle H(M_{i}))\rangle }_{i}+H\left(E_{D}\right)\\ & =\underset{Determinism}{\frac{1}{n}\sum_{i,j}M_{ij}\log_{2}\left(M_{ij}\right)}+\underset{Non-degeneracy}{\sum_{j}\left[-\frac{\sum_{l}M_{lj}}{n}\log_{2}\left(\frac{\sum_{l}M_{lj}}{n}\right)\right]}. \end{array} \end{array}$$

Determinism measures how reliably $x_{t}$ leads to the future state $x_{t+1}$ of the system. Non-degeneracy is the opposite of degeneracy. Degeneracy measures to what degree there is deterministic convergence (not due to noise) from other states onto the future states $x_{t+1}$ specified by $x_{t}$; degeneracy refers to multiple ways of deterministically achieving the same effect and non-degeneracy is opposite to it. We optimized Hoel’s method [22] in which EI=determinism−degeneracy by replacing subtracting degeneracy with adding non-degeneracy, making the calculation commutative. Then, determinism and non-degeneracy can be calculated separately and added together to obtain EI.

Further, we can generalize EI to a continuous state space. We should replace summations with integrals and change the intervened distribution of $x_{t}$ from a discrete equal probability distribution $P(x_{t})=1/n$ to a continuous uniform distribution $U({\left[-L/2,L/2\right]}^{n})$ in which the probability density function is $p\left(x_{t}\right)=1/L^{n}$. Since the intervened distribution cannot be defined on an infinite space such as $R^{n}$, we set the intervention on a limited sub-region of $R^{n}$, which is ${\left[-L/2,L/2\right]}^{n}$, where *L* is a hyperparameter with a value being at least larger than the maximum of $x_{t}$ within finite time steps. Barnett and colleagues introduced a direct link between linear modeling and all the information theoretic measures [52]. Although their model is similar to a stochastic iterative system, it is based on Granger causality and there is no intervention.

We can refer to Hoel’s framework [22,32] to derive an expression for the effective information of a linear stochastic iteration system (Equation (1)). Actually, according to Equation (1), the conditional distribution of $x_{t+1}$ under given $x_{t}$ is a Gaussian distribution, $p\left(x_{t+1}|x_{t}\right)=N\left(Ax_{t},\Sigma \right)$. Therefore, we can compute the EI for this Gaussian distribution [43] as $EI=I(x_{t+1},x_{t}|do\left(x_{t}∼U\right))$, drawing an analogy to mutual information and information entropy within a Gaussian distribution [53] as

(14) $$\begin{array}{c} EI\left(A,\Sigma \right)=\ln\frac{\left|\det\left(A\right)\right|L^{n}}{{\left(2\pi e\right)}^{\frac{n}{2}}\det{\left(\Sigma \right)}^{\frac{1}{2}}}. \end{array}$$

It is particularly emphasized that when n=1, $A=a,\Sigma =\sigma^{2}$ and $J\left(a,\sigma^{2}\right)=EI\left(a,\sigma^{2}\right)$, the effective information can be expressed as

(15) $$\begin{array}{c} \begin{array}{cc} EI(a,\sigma^{2}) & =\ln\frac{aL}{\sqrt{2\pi e}\sigma }, \end{array} \end{array}$$

which is the simplest form of the effective information of within a Gaussian distribution.

Similar to EI(M) of discrete systems, EI(A,Σ) of continuous systems can also be decomposed into two terms, determinism $-{\langle H(p\left(x_{t+1}|x_{t}\right))\rangle }_{x_{t}}$ and non-degeneracy $H(E_{D}\left(x_{t+1}\right))$ as

(16) $$\begin{array}{c} \begin{array}{cc} EI(A,\Sigma ) & =-{\langle H(p\left(x_{t+1}|x_{t}\right))\rangle }_{x_{t}}+H\left(E_{D}\left(x_{t+1}\right)\right)\\ & =\underset{Determinism}{-\ln\left[{\left(2\pi e\right)}^{\frac{n}{2}}\det{\left(\Sigma \right)}^{\frac{1}{2}}\right]}+\underset{Non-degeneracy}{\ln\left(\left|det\left(A\right)\right|L^{n}\right)}. \end{array} \end{array}$$

The higher the determinism or the non-degeneracy, the stronger the causal effect of the stochastic iteration system [22]. The specific derivation process can refer to the calculation process in Appendix A.

Since the calculation of EI involves multiple integrals, $L^{n}$ appears in the expression of EI. When the value of *L* is high, the value of EI becomes higher after *n* powers of *L*. Therefore, we stipulate that when calculating causal emergence, the EI in different dimensions is dimensionally averaged to eliminate the impact of power growth of *L*. Another reason for taking the average on the dimension is that, for the comparison of EI from different dimensions, dimension averaged EI can eliminate *L*, which is shown in Section 3.2. Here, in Theorem 4, we define a new index that is effective information for stochastic iterating systems (J).

**Definition** **4.** *(Effective information for stochastic iterating systems): For the linear stochastic iteration systems like Equation (1), the effective information of the dynamical system is calculated as*

(17) $$\begin{array}{c} J\left(A,\Sigma \right)\equiv \frac{EI(A,\Sigma )}{n}=\frac{1}{n}\ln\frac{\left|\det\left(A\right)\right|L^{n}}{{\left(2\pi e\right)}^{\frac{n}{2}}\det{\left(\Sigma \right)}^{\frac{1}{2}}}=\ln\frac{{\left|\det\left(A\right)\right|}^{\frac{1}{n}}L}{{\left(2\pi e\right)}^{\frac{1}{2}}\det{\left(\Sigma \right)}^{\frac{1}{2n}}} \end{array}$$

*where det(·) represents the determinant value corresponding to a matrix ·, |·| represents the absolute value of ·, and L represents the interval size of the do(·) intervention.*

*The effective information J can also be decomposed into two parts, determinism and non-degeneracy, as*

(18) $$\begin{array}{c} \begin{array}{cc} J(A,\Sigma ) & =\underset{Determinism}{-\ln\left[{\left(2\pi e\right)}^{\frac{1}{2}}\det{\left(\Sigma \right)}^{\frac{1}{2n}}\right]}+\underset{Non-degeneracy}{\ln\left({\left|det\left(A\right)\right|}^{\frac{1}{n}}L\right)}. \end{array} \end{array}$$

For calculation process of Definition 4, refer to Appendix A. J(A,Σ) can be used to measure the causal effects of micro dynamics and macro dynamics and then be used to calculate causal emergence. It should be noted that the scope *L* of do intervention in macro-state dynamics EI is related to $W\in R^{n\times k}$. Therefore, we need to limit the range of *W* to ensure the do intervention range of macro-state $y_{t}\in R^{k}$ and micro-state $x_{t}\in R^{n}$ as $do\left(y_{t}\right)∼U\left({\left[-L/2,L/2\right]}^{k}\right)$ and $do\left(x_{t}\right)∼U\left({\left[-L/2,L/2\right]}^{n}\right)$, on which we will conduct more analysis in Section 3.4.

### 3.2. Causal Emergence

After obtaining effective information for stochastic iterating systems J at different dimensions *k* of variables $x_{t}$, we still have a problem to solve. Except for the influence of indices, the value of hyperparameter *L* itself, which is artificially assumed, also has a great influence on the results of J. This hyperparameter can be eliminated by calculating causal emergence for stochastic iterating systems as Definition 5.

**Definition** **5.** *(Causal emergence for stochastic iterating systems) Hyperparameter L can be eliminated by subtraction as*

(19) $$\begin{array}{c} \Delta J=J_{M}-J_{m}, \end{array}$$

*where $J_{m}\equiv J\left(A,\Sigma \right)$ is the EI for the micro-dynamics (Equation (1), $J_{M}\equiv J\left(A_{M},\Sigma_{M}\right)$ is the EI for the macro-dynamics (Equation (8)), and ΔJ is the degree of causal emergence for stochastic iterating systems.*

Through the calculations in Appendix A, we obtain the following theorem. In this theorem, we not only calculate the analytical solution for causal emergence but also ensure that the degree of the analytical solution is only related to the parameters of stochastic iterating systems without the influence of *L*.

**Theorem** **1.** *(Analytical solution for causal emergence): For the linear stochastic iteration systems like Equation (1), causal emergence of the system after coarse-graining $y_{t}=\phi \left(x_{t}\right)=Wx_{t}$, $W\in R^{k\times n}$, is calculated as*

(20) $$\begin{array}{c} \Delta J=\underset{Non-degeneracyEmergence}{\ln\frac{|\det\left(WAW^{†}\right){|}^{\frac{1}{k}}}{{\left|\det\left(A\right)\right|}^{\frac{1}{n}}}}+\underset{DeterminismEmergence}{\ln\frac{{\left|\det\left(\Sigma \right)\right|}^{\frac{1}{2n}}}{|\det\left(W\Sigma W^{T}\right){|}^{\frac{1}{2k}}}} \end{array}$$

*$W\in R^{k\times n}$, $x_{t}\in R^{n}$, $y_{t}\in R^{k}$. Causal Emergence can also be divided into two terms, and we name them as non-degeneracy emergence and determinism emergence separately. Both terms depend on the coarse-grained results of the overall derivative and noise indicators, respectively.*

The premise for Theorem 1 to hold is that both macro and micro states can be represented by *L* as the range of values, and the premise is that the two cannot differ by too much order of magnitude. When the intervention interval size for each dimension of micro- and macro-variables is consistent and equal to *L*, *L* can be directly eliminated when calculating causal emergence, so ΔJ is independent with *L*.

### 3.3. Some Bounds Related with Causal Emergence

After inferring the calculation method of ΔJ, we can determine whether there is causal emergence in the macro states after coarse-graining. Next, we will find an optimal matrix *W* to maximize the degree of causal emergence ΔJ [22,43]. To solve this problem, we will firstly discuss the upper bounds related with the two terms, the determinism emergence and non-degeneracy emergence, in Equation (20) separately. Here we have the lemmas below.

The first lemma is to analyze non-degeneracy emergence by optimizing $A_{M}=WAW^{†}$.

**Lemma** **1.** *For matrix A, its eigenvalues can be real numbers or complex numbers, while the range of absolute values (modulus) for $\left|\det(WAW^{†})\right|$ is bounded:*

(21) $$\begin{array}{c} 0\leq \left|\det(WAW^{†})\right|\leq \prod_{i=1}^{k}\left|\lambda_{i}\right|, \end{array}$$

*where $A\in R^{n\times n}$, $W\in R^{k\times n}$, $W^{†}\in R^{n\times k}$ is the Moore–Penrose generalized inverse matrix of W, and k is the rank of the matrix W.*

*(1)*  *When the eigenvalues are all real numbers, $|\lambda_{1}\left|\geq \right|\lambda_{2}\left|\geq \cdots \geq \right|\lambda_{n}|\geq 0$ are n absolute values of eigenvalues of matrix A, sorted from largest to smallest, $\lambda_{1},\lambda_{2},\ldots ,\lambda_{n}\in R$.*
*(2)*  *When the eigenvalues contain imaginary numbers, |·| represents the modulus of complex numbers. Note that since the eigenvalues of imaginary numbers appear together with their conjugate complex numbers, both eigenvalues must have equal moduli and must be discarded or retained simultaneously.*
*(3)*  *When A is a symmetric matrix and A>0 is a positive definite matrix, since $\lambda_{1}\geq \lambda_{2}\geq \cdots \geq \lambda_{n}\geq 0$, we can just write the equation as*
(22) $$\begin{array}{c} \prod_{i=n-k+1}^{n}\lambda_{i}\leq \det\left(WAW^{†}\right)\leq \prod_{i=1}^{k}\lambda_{i}. \end{array}$$

**Figure 1:**
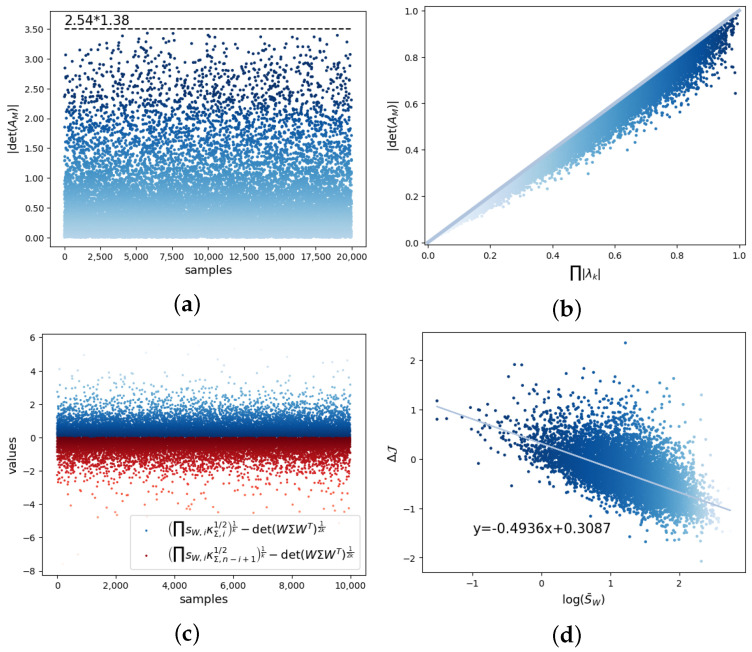
Experimental results of the simulation of the lemma and the theorem presented in Section 3. (**a**) For matrix *A* with eigenvalues of $\lambda_{1}=2.540,\;\lambda_{2}=1.380,\;\lambda_{3}=-0.4899,\;\lambda_{4}=0.1149$, we randomly generate matrix *W*. We can see that there is an upper bound $\lambda_{1}\lambda_{2}$ on the corresponding $\det(A_{M})$ value of each *W*. (**b**) If *A* is also randomly generated, we can see the scatter plot that conforms to Lemma 1. (**c**) We validate the inequality using randomly generated Σ and *W*. We can control the range of $\det(W\Sigma W^{T})$ by adjusting the magnitude of the singular value of *W*. (**d**) The smaller the mean singular value of *W* we generate, the greater the degree of causal emergence ΔJ.

For proof of the lemma, refer to in Appendix B. In addition, we can verify this lemma through numerical simulations. For example, for matrix *A* with eigenvalues of $\lambda_{1}=2.540,\;\lambda_{2}=1.380,\;\lambda_{3}=-0.4899,\;\lambda_{4}=0.1149$, we randomly generate the elements of the matrix *W*. From Figure 1a, we can see that there is an upper bound $\lambda_{1}\lambda_{2}$ on the corresponding $\det(A_{M})$ value of each *W*. If *A* is also randomly generated, we can see that all the scatter points are below the diagonal line y=x in Figure 1b, which means Lemma 1 is satisfied.

After the lemma regarding $|\det\left(A_{M}\right)|=|\det\left(WAW^{†}\right)|$, the next lemma regards $\det\left(\Sigma_{M}\right)=\det\left(W\Sigma W^{T}\right)$, which is related to the determinism emergence.

**Lemma** **2.** *We define the singular values of W as $s_{W,1}\geq s_{W,2}\geq \cdots \geq s_{W,k}$, then $\det(W\Sigma W^{T})$ satisfies*

(23) $$\begin{array}{c} {\left(\prod_{i=1}^{k}s_{W,i}\kappa_{n-i+1}^{\frac{1}{2}}\right)}^{\frac{1}{k}}\leq \det{\left(W\Sigma W^{T}\right)}^{\frac{1}{2k}}\leq {\left(\prod_{i=1}^{k}s_{W,i}\kappa_{i}^{\frac{1}{2}}\right)}^{\frac{1}{k}}, \end{array}$$

*where $\kappa_{1}\geq \kappa_{2}\geq \cdots \geq \kappa_{n}$ are the eigenvalues of Σ. When $\Sigma =\sigma^{2}I$, the equal sign holds and*

(24) $$\begin{array}{c} \det{\left(W\Sigma W^{T}\right)}^{\frac{1}{2k}}=\sigma {\left(\prod_{i=1}^{k}s_{W,i}\right)}^{\frac{1}{k}}. \end{array}$$

In Figure 1c, we validate the inequalities using randomly generated Σ and *W*. Therefore, we can control the range of $\det(W\Sigma W^{T})$ by adjusting the magnitude of the singular values of *W*. We further can adjust the magnitude of the singular value of *W* to weaken the noise and enhance the causal emergence, as we can see in Lemma 2. As shown in Figure 1d, the smaller the mean singular values of *W* we generate, the greater the degree of causal emergence ΔJ.

### 3.4. Causal Emergence Maximization under Given Information Loss

In this subsection, we will officially discuss the problem of causal emergence maximization. Before proceeding, we will introduce a condition on uncertainty elimination to constrain *W*, such that zeros in the denominator are excluded.

#### 3.4.1. The Constraint on the Uncertainty Elimination by Coarse-Graining

According to Lemma 2, when the singular value of *W*, $s_{W,1}\to 0$, $\det(W\Sigma W^{T})\to 0$. This means if $\phi \left(x_{t}\right)=Wx_{t}$ is a completely unconstrained map, as $do\left(y_{t}\right)∼U\left({\left[-L/2,L/2\right]}^{k}\right)$ and $do\left(x_{t}\right)∼U\left({\left[-L/2,L/2\right]}^{n}\right)$, the values of $x_{t}$ and the noise $\epsilon_{t}$ may be scaled in an unbounded manner and the information entropy $H(p\left(y_{t+1}|y_{t}\right))\to -\infty$, leading to $y_{t+1}$ having an excessively narrow range. To prevent the macro variables from being excessively compressed by the coarse-graining map, which may result in excessive information content, it is essential to impose the following restriction,

(25) $$\begin{array}{c} \frac{1}{n}H\left(p\left(x_{t+1}|x_{t}\right)\right)-\frac{1}{k}H\left(p\left(y_{t+1}|y_{t}\right)\right)\leq \eta , \end{array}$$

where *n* and *k* are the dimensionalities of micro- and macro-variables, and η>0 is a given constant, and $H\left(p\left(x_{t+1}|x_{t}\right)\right)=\left(n/2\right)\ln\left(2\pi e\right)+\left(1/2\right)\ln\left(\det\left(\Sigma \right)\right)$ and $H\left(p\left(y_{t+1}|y_{t}\right)\right)=\left(k/2\right)\ln\left(2\pi e\right)+\left(1/2\right)\ln\left(\det\left(W\Sigma W^{T}\right)\right)$. The inequality can be understood as the dimension averaged uncertainty eliminated (or information gained) by the coarse-graining strategy under a given threshold η. According to the inequality (25), we can further get that

(26) $$\begin{array}{c} \det{\left(W\Sigma W^{T}\right)}^{\frac{1}{k}}\geq \epsilon \det{\left(\Sigma \right)}^{\frac{1}{n}}, \end{array}$$

where $W^{T}$ represents the transpose matrix of matrix *W* and ϵ=exp(−2η). The covariance matrix affects the range of values of random variables; the limitations here can also ensure the effectiveness of the do intervention scope *L* mentioned in Section 3.1. It will be clear in the subsequent sections that this constraint could also significantly influence the scope of causal emergence.

With the help of Lemmas 1 and 2, we can obtain the optimal solution for causal emergence $\Delta J^{*}$.

**Theorem** **2.** *For linear stochastic iteration systems like Equation (1), after coarse-graining $y_{t}=\phi \left(x_{t}\right)=Wx_{t}$, $W\in R^{k\times n}$, under the constraint of Equation (26), the maximum of the degree of causal emergence that the system can achieve is*

(27) $$\begin{array}{c} \Delta J^{*}=\frac{1}{k}\sum_{i=1}^{k}\ln|\lambda_{i}|-\frac{1}{n}\sum_{i=1}^{n}\ln\left|\lambda_{i}\right|+\eta , \end{array}$$

*where $|\lambda_{1}\left|\geq \right|\lambda_{2}\left|\geq \cdots \geq \right|\lambda_{n}|\geq 0$ are n modulus of eigenvalues of matrix A sorted from the largest to the smallest, $\lambda_{1},\lambda_{2},\ldots ,\lambda_{n}\in C$.*

**Proof.**  We will discuss the maximization of causal emergence from determinism emergence and non-degeneracy emergence separately.

(1) Non-degeneracy emergence ($\Delta J_{1}$)
  Based on Theorem 1, we know that
  (28) $$\begin{array}{c} \begin{array}{cc} \Delta J_{1} & =\ln\frac{|\det\left(WAW^{†}\right){|}^{\frac{1}{k}}}{{\left|\det\left(A\right)\right|}^{\frac{1}{n}}}\leq \ln\frac{{\left(\prod_{i=1}^{k}\left|\lambda_{i}\right|\right)}^{\frac{1}{k}}}{{\left(\prod_{i=1}^{n}\left|\lambda_{i}\right|\right)}^{\frac{1}{n}}}=\frac{1}{k}\sum_{i=1}^{k}\ln|\lambda_{i}|-\frac{1}{n}\sum_{i=1}^{n}\ln\left|\lambda_{i}\right|. \end{array} \end{array}$$
  If and only if the maximum *k* eigenvalues are preserved, the equality holds. At this point, $A_{M}=WAW^{†}=\left(WV\right)\Lambda {\left(WV\right)}^{†}=\tilde{W}\Lambda {\tilde{W}}^{†}$, $\Lambda =\mathrm{diag}\left(\lambda_{1},\ldots ,\lambda_{n}\right)\in R^{n\times n}$; it is satisfied that $\tilde{W}=\left({\tilde{W}}_{k},O_{k\times \left(n-k\right)}\right)$, ${\tilde{W}}_{k}\in R^{k\times k}$ can be any invertible matrix. $V=(v_{1},\ldots ,v_{n})$; $v_{i}$ is the eigenvector corresponding to the eigenvalues $\lambda_{i}$ of matrix *A*, i=1,…,n, $|\lambda_{1}\left|\geq \cdots \geq \right|\lambda_{n}|$. Since $\tilde{W}=WV$, we can obtain the expression for *W* as
  (29) $$\begin{array}{c} W=\left({\tilde{W}}_{k},O_{k\times \left(n-k\right)}\right)V^{-1}. \end{array}$$
(2) Determinism emergence ($\Delta J_{2}$)
  Based on Equation (20) and the inequalities (23), (25), and (26), we know that
  (30) $$\begin{array}{c} \begin{array}{cc} \Delta J_{2} & =\ln\frac{{\left|\det\left(\Sigma \right)\right|}^{\frac{1}{2n}}}{|\det\left(W\Sigma W^{T}\right){|}^{\frac{1}{2k}}}=\frac{1}{2}\ln\frac{{\left|\det\left(\Sigma \right)\right|}^{\frac{1}{n}}}{|\det\left(W\Sigma W^{T}\right){|}^{\frac{1}{k}}}\\ & \leq \frac{1}{2}\ln\frac{{\left|\det\left(\Sigma \right)\right|}^{\frac{1}{n}}}{{\left|\det\left(\Sigma \right)\right|}^{\frac{1}{n}}\epsilon }=\frac{1}{2}\ln\frac{1}{\epsilon }=\eta \end{array}. \end{array}$$
  When the information loss reaches the upper bound, the determinism emergence reaches the maximum. Determinism is enhanced while ensuring that randomness is not excessively reduced.

By taking the maximum value of $\Delta J_{1}$ and $\Delta J_{2}$, we can obtain

(31) $$\begin{array}{c} \begin{array}{cc} \Delta J^{*} & =\Delta J_{1}^{*}+\Delta J_{2}^{*}=\ln\frac{{\left(\prod_{i=1}^{k}\left|\lambda_{i}\right|\right)}^{\frac{1}{k}}}{{\left(\prod_{i=1}^{n}\left|\lambda_{i}\right|\right)}^{\frac{1}{n}}}+\frac{1}{2}\ln\frac{1}{\epsilon }=\frac{1}{k}\sum_{i=1}^{k}\ln|\lambda_{i}|-\frac{1}{n}\sum_{i=1}^{n}\ln\left|\lambda_{i}\right|+\eta \end{array}. \end{array}$$

□

Suppose the solution set of the optimal coarse-grained matrix $W^{*}$, which satisfies $\Delta J\left(W^{*}\right)=\Delta J^{*}$, is $W^{*}$. Although it is difficult for us to find all the elements in set $W^{*}$, we will provide methods for calculating special solutions for different types of matrices in later sections.

#### 3.4.2. Optimal Solution Set $W^{*}$

After optimizing determinism emergence and non-degeneracy emergence, we can find two solution sets corresponding to the optimal solutions. Thus, the intersection of the two solution sets is the solution set of *W* corresponding to the maximum degree of causal emergence.

**Corollary** **1.** *When $\varepsilon_{t}∼N\left(0,\Sigma \right)$ and the degree of causal emergence reaches its maximum value, W needs to satisfy*

(32) $$\begin{array}{c} \left\{\begin{array}{c} WV=({\tilde{W}}_{k},O_{k\times \left(n-k\right)}),\\ \det{\left(W\Sigma W^{T}\right)}^{\frac{1}{k}}=\epsilon \det{\left(\Sigma \right)}^{\frac{1}{n}}, \end{array}\right. \end{array}$$

in which $V=(v_{1},\ldots ,v_{n})$; $v_{i}$ is the eigenvector corresponding to the eigenvalues $\lambda_{i}$ of matrix A, i=1,…,n, $|\lambda_{1}\left|\geq \cdots \geq \right|\lambda_{n}|$. ${\tilde{W}}_{k}\in R^{k\times k}$ can be any invertible matrix. The solution set of the optimal coarse-grained matrix $W^{*}$ satisfying $\Delta J\left(W^{*}\right)=\Delta J^{*}$ as ϵ=exp(−2η) is

(33) $$\begin{array}{c} W^{*}=\left\{W|W=\left({\tilde{W}}_{k},O_{k\times \left(n-k\right)}\right)V^{-1},\det\left(W\Sigma W^{T}\right)=\epsilon \det{\left(\Sigma \right)}^{\frac{k}{n}}\right\}. \end{array}$$

We can find the proof process in the previous Section 3.4.

#### 3.4.3. A Case Study of $W^{*}$

To understand the physical meaning of the solution set, we can use a simple example in the three-dimensional space to visualize our optimal solution set. In specific cases, when k=2,n=3, the projection of *W* in three-dimensional space is a circle. In this case, matrix $W={\left(w_{1}^{T},w_{2}^{T}\right)}^{T}\in R^{2\times 3}$ can be split into two row-vectors, $w_{i}=\left(w_{i1},w_{i2},w_{i3}\right),i=1,2$, in $R^{3}$, The eigenvector matrix $V=\left(v_{1},v_{2},v_{3}\right)\in R^{3\times 3}$ can be regarded as the combining of three vectors, $v_{j}={\left(v_{1j},v_{2j},v_{3j}\right)}^{T}\in R^{3},j=1,2,3$. To visualize the solution set of *W*, we introduce the following case as ϵ=exp(−2η).

**Example** **1.** *In order to present the results more intuitively, we specify $w_{1}w_{2}^{T}=0$, $\Sigma =\sigma^{2}I_{3}$. According to Equation (33), we can get*

(34) $$\begin{array}{c} \left\{\begin{array}{c} w_{1}v_{3}=0,\\ w_{2}v_{3}=0,\\ \left(w_{1}w_{1}^{T}\right)\left(w_{2}w_{2}^{T}\right)=\epsilon \end{array}\right.. \end{array}$$

*Although $W\in R^{2\times 3}$ is in a six-dimensional space, we can obtain the solution set of $w_{1}$ for specified $w_{2}$,*

(35) $$\begin{array}{c} \left\{\begin{array}{c} w_{11}v_{13}+w_{12}v_{23}+w_{13}v_{33}=0\\ w_{11}^{2}+w_{12}^{2}+w_{13}^{2}=R^{2} \end{array}\right., \end{array}$$

*in which $R^{2}=\epsilon /\left(w_{2}w_{2}^{T}\right)$; R is the radius of the sphere. Similarly, we can also obtain the range of values for $w_{2}$.*

**Proof.**  To meet the conditions $WV=\left(Wv_{1},Wv_{2},Wv_{3}\right)=\left({\tilde{W}}_{k},O_{k\times \left(n-k\right)}\right)$, we only need to specify $w_{1}v_{3}=w2v_{3}=0$. This means that $w_{1}$ and $w_{2}$ are contained in the plane perpendicular to $v_{3}$ and passing through the origin, which is $w_{11}v_{13}+w_{12}v_{23}+w_{13}v_{33}=0$. When $w_{1}$ and $w_{2}$ are in this plane, the non-degeneracy emergence $\Delta J_{1}$ reaches its minimum value.

For determinism emergence $\Delta J_{2}$,

(36) $$\begin{array}{c} \begin{array}{cc} \det\left(W\Sigma W^{T}\right) & =\left(w_{1}\Sigma w_{1}^{T}\right)\left(w_{2}\Sigma w_{2}^{T}\right)-\left(w_{1}\Sigma w_{2}^{T}\right)\left(w_{2}\Sigma w_{1}^{T}\right)\\ {\;}_{\left(\Sigma =\sigma^{2}I_{3}\right)} & =\sigma^{4}\left[\left(w_{1}w_{1}^{T}\right)\left(w_{2}w_{2}^{T}\right)-\left(w_{1}w_{2}^{T}\right)\left(w_{2}w_{1}^{T}\right)\right]\\ {\;}_{w_{1}w_{2}^{T}=0} & =\sigma^{4}\left[\left(w_{1}w_{1}^{T}\right)\left(w_{2}w_{2}^{T}\right)\right]=\epsilon \det{\left(\Sigma \right)}^{\frac{k}{n}}\\ & =\epsilon \sigma^{4} \end{array}. \end{array}$$

When $w_{2}$ is fixed, the solution set of $w_{1}$ is a sphere in three-dimensional space, $w_{11}^{2}+w_{12}^{2}+w_{13}^{2}=R^{2},R^{2}=\epsilon /\left(w_{2}w_{2}^{T}\right)$. If we fix $w_{1}$, the result is the same. □

From this example, we can derive the following proposition:

**Proposition** **1.** In three-dimensional space $R^{3}$, if the noise is a white noise sequence $\Sigma =\sigma^{2}I_{3}$ and $w_{1}$ and $w_{2}$ are perpendicular to each other, when causal emergence $\Delta J\left(W^{*}\right)=\Delta J^{*}$, the solution set of $w_{i}$ for i=1,2 is the intersection of a plane and a sphere in the space, which is a circle.

**Figure 2:**
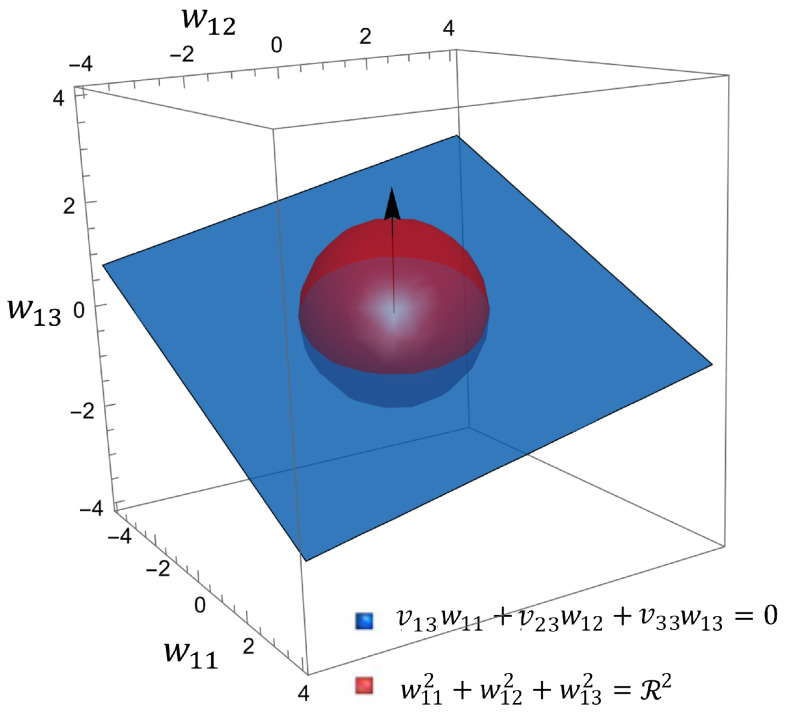
The visualization of the set for the optimal solutions of the coarse-graining strategy *W*. When $w_{1}w_{2}^{T}=0$, $\Sigma =\sigma^{2}I_{3}$. Although $W\in R^{2\times 3}$ is in a six-dimensional space, we can draw the range of values for $w_{1}$ while limiting $w_{2}$, $w_{11}v_{13}+w_{12}v_{23}+w_{13}v_{33}=0$ and $w_{11}^{2}+w_{12}^{2}+w_{13}^{2}=R^{2},$ in which $R^{2}=\epsilon /\left(w_{2}w_{2}^{T}\right)$. When causal emergence $\Delta J\left(W^{*}\right)=\Delta J^{*}$, the solution set of $w_{i}$ is the intersection of a plane (blue) and a sphere (red) in 3D space $R^{3}$, which is a circle.

In Figure 2, we visualize the solution set of $w_{1}=\left(w_{11},w_{12},w_{13}\right)$. At the same time, we can extend the scope of application of Example 1 for the general form of covariance matrix Σ; the solution set becomes an ellipsoid. If $w_{1}w_{2}^{T}\neq 0$, then the spherical or ellipsoidal surface undergoes translation. Therefore, the final solution sets for $w_{1}$ and $w_{2}$ are the intersection lines of ellipsoids or spheres with planes. Two spatial curves form the solution set of *W*. Therefore, we can obtain the proposition below.

**Proposition** **2.** *In three-dimensional space $R^{3}$, Σ>0 the coarse-graining parameter matrix W can be regarded as composed of k row vectors $w_{i}$, k=1,2,3, i=1,…,k; the solution space of each vector $w_{i}$ is the intersection of a plane and an ellipsoid, which is an ellipse.*

With a visualized solution set in three-dimensional space, we can infer the optimal solution of *W* in high-dimensional space.

**Proposition** **3.** For any n>1, a coarse-graining matrix $W\in R^{k\times n}$ can be split into k row vectors $w_{i}\in R^{n}$ in the same way, and the solution set of $W^{*}$ can be understood as the intersection of hyperplanes and hyperellipsoids in an $R^{n}$.

Therefore, after obtaining the relevant parameters of dynamics, we first derive the expression for a high-dimensional ellipse, where any point on the high-dimensional ellipse can maximize the causal emergence. At the same time, when the model is more complex, we can also choose to find points close to the ellipse, even if it is not the optimal solution, which can increase the likelihood of causal emergence.

### 3.5. The Conditions for Causal Emergence

After understanding the solution method for the maximum causal emergence of the system, we need to analyze the conditions under which causal emergence occurs as ΔJ>0. Two scenarios are being examined: the presence of causal emergence in typical situations and its manifestation under optimized conditions.

First, in general cases, causal emergence ΔJ is determined by two factors: non-degeneracy $\Delta J_{1}$ and determinism $\Delta J_{2}$. The sufficient condition for ΔJ>0 is that $\Delta J_{1},\Delta J_{2}>0$ at the same time.

The prerequisite for $\Delta J_{1}>0$ is that there is a significant difference between the eigenvalues $\lambda_{1}\geq \cdots \geq \lambda_{n}$ of parameter matrix $A\in R^{n\times n}$. Assuming the macro-state $y_{t}\in R^{k}$, the greater the difference between $\lambda_{1},\ldots ,\lambda_{k}$ and $\lambda_{k+1},\ldots ,\lambda_{n}$, the more obvious the causal emergence. The best condition is that $\lambda_{k}\gg \lambda_{k+1}$; if the latter n−k term is discarded, there will be a significant causal emergence.

The condition for $\Delta J_{2}>0$ is that there is significant randomness in the system as Σ is a positive definite matrix and $H(p\left(x_{t+1}|x_{t}\right))>-\infty$ when $do\left(y_{t}\right)∼U\left({\left[-L/2,L/2\right]}^{k}\right)$ and $do\left(x_{t}\right)∼U\left({\left[-L/2,L/2\right]}^{n}\right)$. If Σ=0 and $H(p\left(x_{t+1}|x_{t}\right))=-\infty$, as there is no random noise in the system, effective information is also invalid. In theory, the larger the system noise, the larger the space that can be compressed during coarse-graining, and the stronger the causal emergence can appear.

Second, when causal emergence reaches its optimal solution, we can also determine the condition for ΔJ>0 based on the result of $\Delta J^{*}$. From Equation (31), we can see that when

(37) $$\begin{array}{c} \begin{array}{c} \frac{1}{n}\sum_{i=1}^{n}\ln|\lambda_{i}|-\frac{1}{k}\sum_{i=1}^{k}\ln\left|\lambda_{i}\right|<\eta \end{array} \end{array}$$

as the difference of the average singular value of *A* to the average singular value of $A_{M}$ after taking the logarithm can be smaller than the bound of the information entropy η we specify, $\Delta J^{*}>0$. If this result holds, we can assert that there must exist a *W* to make ΔJ>0.

## 4. Results

After understanding how to optimize causal emergence, we can use the theorems we derived to analyze several cases of linear stochastic iteration systems. We will attempt to search for the maximum of causal emergence in systems with known dynamics. We provide three cases, focusing on the applications of determinism emergence, non-degeneracy emergence, and the manifestation of causal emergence in $R^{3}$ space.

### 4.1. Random Walk

Our first case is random walk [54], and our analysis focuses on the noise $\varepsilon_{t}$ and the covariance matrix Σ, which mainly impact determinism emergence. The random walk model is a mathematical model used to describe the random movement of objects in a specific space in which a walker moves between a series of locations, with the direction and distance of each movement being random. This model can study various phenomena, such as changes in asset prices in financial markets, the diffusion of particles in fluids, etc. In the random walk model, the walker can only move a certain distance to the left or right each time, and the distance obeys a normal distribution. When multiple wanderers coexist, a changing parameter matrix can be formed as an identity matrix, $A=I_{n}$,

(38) $$\begin{array}{c} x_{t+1}=x_{t}+\varepsilon_{t},\varepsilon_{t}∼N\left(0,\Sigma \right), \end{array}$$

which forms a linear stochastic series mainly affected by noise sequences $\varepsilon_{t}$.

We set an example as n=4, k=1, while

(39) $$\begin{array}{c} \begin{array}{c} \Sigma =\left(\begin{array}{cccc} 0.4782 & -0.1967 & -0.0287 & 0.0419\\ -0.1967 & 0.6711 & 0.0233 & -0.1067\\ -0.0287 & 0.0233 & 0.3154 & 0.0738\\ 0.0419 & -0.1067 & 0.0738 & 0.4211 \end{array}\right). \end{array} \end{array}$$

**Figure 3:**
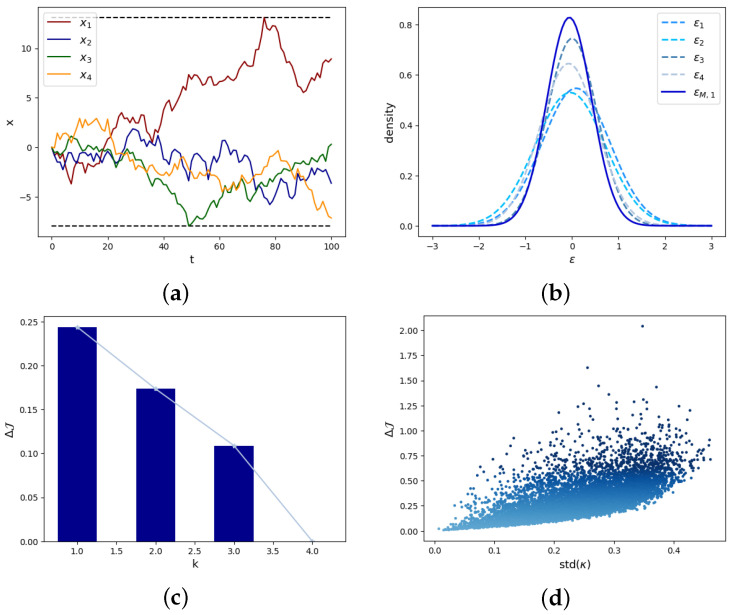
Experimental results for random walks model. (**a**) The trajectories $x_{t}$ of a random walker in different dimensions. (**b**) The probability density functions for the four dimensions of micro-state noises $\varepsilon_{t}$ and the macro-state noise $\varepsilon_{M,t}$. (**c**) The degree of causal emergence under different macro-state dimensions *k*. Under the condition that the singular values of *W* are all 1, the higher *k*, the stronger the uncertainty of the system, and the smaller the degree of causal emergence. (**d**) ΔJ and std(κ). The larger the variance of the eigenvalues $\kappa_{i}$ of Σ, i=1,…,n, the more likely the occurrences of causal emergence with higher degrees.

The motion trend of each dimension of $x_{t}$ is shown in Figure 3a. From Equation (26), we already know that the ratio between $\det{\left(W\Sigma W^{T}\right)}^{1/k}$ and $\det{\left(\Sigma \right)}^{1/n}$ should be greater than the lower bound ϵ. The constant for uncertainty loss is η=0.3466 and the corresponding ϵ=0.5. This value being selected guarantees that when the singular values of *W* are all 1, the determinism emergence $\Delta J_{2}=0.2439>0$ as W=(−0.0819,0.1432,−0.8421,0.5135) satisfies $\det{\left(W\Sigma W^{T}\right)}^{\frac{1}{k}}/\det{\left(\Sigma \right)}^{\frac{1}{n}}=0.614>\epsilon$.

We have plotted probability density graphs for the four dimensions of micro-state noise $\varepsilon_{t}$ and macro-state noise $\varepsilon_{M,t}$ in Figure 3b, respectively. We can see that the variance of macro-state is smaller than that of micro-state, so the determinism emergence of macro-state dynamical systems is larger. Meanwhile, in Figure 3c we can see that under the condition that the singular values of *W* are all 1, the higher *k*, the stronger the uncertainty of the system, and the smaller the degree of causal emergence.

Reducing the average noise of the system and increasing its determinism is an important condition for the occurrence of causal emergence. The main reason for this is that we can reduce the dimensions with weaker determinism. The premise for this phenomenon is that there are significant differences in noise between different dimensions. As shown in Figure 3d, we randomly generate Σ and *W* and calculate the value of ΔJ. We found that the larger the variance of the eigenvalues $\kappa_{i}$ of Σ, i=1,…,n, the more likely the occurrence of causal emergence.

### 4.2. Heat Dissipation

**Figure 4:**
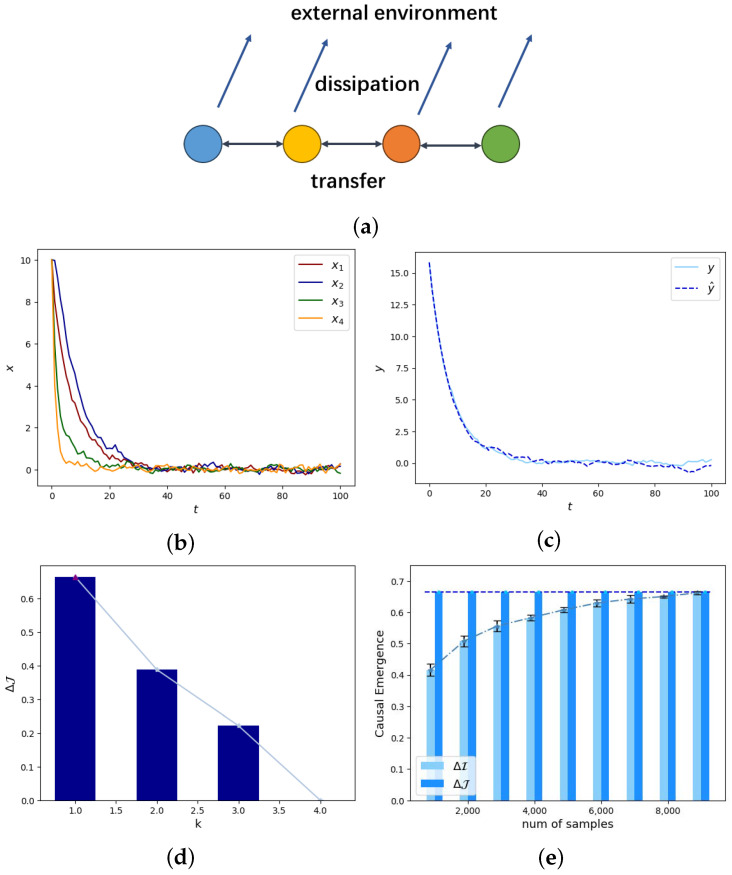
Experimental results for heat dissipation model. (**a**) The illustration of heat transfer and dissipation for a one-dimensional chain with four vertices. (**b**) The micro-state data of temperature at different times *t* for 4 nodes. (**c**) The comparison between the macro-state data directly obtained by coarsening the micro-state data, $y_{t}$, to obtain $y_{t}=Wx_{t}$ for t=0,1,2,…, and ${\hat{y}}_{t}$, which is generated by the macro-state dynamics to iterate the initial macro-state $y_{0}$. (**d**) The comparisons for the degrees of causal emergence under different dimensions of macro-states *k*. when k=1 and ϵ=1, a maximized degree of causal emergence occurs as ΔJ=0.6656. (**e**) The comparison between ΔI and ΔJ. As the sample size increases, the numerical solutions for causal emergence ΔI gradually approach the analytical solutions ΔJ.

The first case focuses on determinism and noise, while the second case focuses on non-degeneracy and parameter matrix *A*. This case is called the discretized [55] heat conduction model [56,57]. In this discretized case, the conduction of heat is mainly reflected in the temperature changes over time [58] at the corresponding observation nodes [59]. We can use matrix *A* to represent the changes in temperature, which includes information on the rate of change. This matrix usually corresponds to a positive definite sparse matrix, which can be used to describe a system’s temperature change process. For example,

(40) $$\begin{array}{c} x_{t+1}=Ax_{t}+\varepsilon_{t},\varepsilon_{t}∼N\left(0,\sigma^{2}I_{n}\right),\sigma =0.01 \end{array}$$

describes the variation of temperature $x_{t}$ over time *t*. When n=4, $x_{t}={\left(x_{1,t},x_{2,t},x_{3,t},x_{4,t}\right)}^{T}$, $x_{1,t}$, $x_{2,t}$, $x_{3,t}$, and $x_{4,t}$ represent the temperatures at four nodes at time *t*, as shown in Figure 4b. The parameter matrix is

(41) $$\begin{array}{c} \begin{array}{c} A=\left(\begin{array}{cccc} 0.6 & 0.2 & 0 & 0\\ 0.2 & 0.7 & 0.1 & 0\\ 0 & 0.1 & 0.4 & 0.1\\ 0 & 0 & 0.1 & 0.3 \end{array}\right), \end{array} \end{array}$$

where diagonal elements represent the rates of heat conservation at each node, and the off-diagonals represent the energy transfer between different nodes, as shown in Figure 4a. Assuming ϵ=1 as η=0, then the uncertainties in both the dynamics at micro- and macro-levels are the same according to Equation (25). As a result, only the effect of non-degeneracy emergence can influence the causal emergence according to Equation (31).

We can simulate the diffusion process by setting the initial state to $x_{0}={\left(10,10,10,10\right)}^{T}$, and we iterate the equation $x_{t+1}=Ax_{t}+\varepsilon_{t}$ for t=1,2,…. The results are shown in Figure 4b. We can directly coarse-grain the micro-state data to obtain $y_{t}=Wx_{t}$ for t=0,1,2,…. At the same time, we can use the macro-state dynamics to iterate the initial macro-state $y_{0}$. In order to distinguish from the previous $y_{t}$ data, we use ${\hat{y}}_{t}$ to represent the data generated by macro-state dynamical iterations. ${\hat{y}}_{0}=y_{0}$ and ${\hat{y}}_{t+1}=WAW^{†}{\hat{y}}_{t}+W\varepsilon_{t}$ for t=0,1,2,…. By comparison in Figure 4c, we can see that the dynamics of $y_{t}$ and ${\hat{y}}_{t}$ are close to each other; macro-state dynamics can represent the dynamic evolution law of data after the coarse-graining.

By reducing the low correlation and dimensions that are difficult to reverse between $x_{t}$ and $x_{t+1}$, non-degeneracy can be increased, resulting in stronger causal emergence. For example, in the case shown in Figure 4d, when k=1, σ=0.01, and η=0 as Shannon entropy of $x_{t}$ and $y_{t}$ remains stable, causal emergence occurs with the degree ΔJ=0.6656, and it is greater than the case when k>1. We can take any element from $W^{*}$; for example, $W=\left(0.5856,0.7910,0.1748,0.03065\right)\in R^{1\times k}$, which satisfies our condition of *W* in Equation (32). The macro-state dynamic parameter matrix is $A_{M}\in R^{1\times 1}$ with an eigenvalue $\lambda_{M,1}=0.8702$. The eigenvalues of the micro-state matrix *A* are $\lambda_{1}=0.8702,\;\lambda_{2}=0.5000,\;\lambda_{3}=0.4000$, and $\lambda_{4}=0.2298$. It can be seen that retaining the maximum eigenvalue will result in significant causal emergence. In physical terms, it can be understood as a region with slower dissipation, and the causal relationship between the temperature at time t+1 and time *t* is stronger, making it more suitable for analyzing the temperature evolution law of the entire system.

To verify our theoretic result about the exact expression for causal emergence, we can compare the obtained analytical solution in Equation (27) with the numerical results, which can be obtained by the following steps. We first randomly generate numeric samples of $x_{t}∼U\left({\left[-1,1\right]}^{n}\right)$ and $y_{t}∼U\left({\left[-1,1\right]}^{k}\right)$, then perform a one-step iteration for micro- and macro-dynamics as $x_{t+1}=Ax_{t}+\varepsilon_{t}$ and $y_{t+1}=A_{M}y_{t}+\varepsilon_{M,t}$, $\varepsilon_{t}∼N_{n}\left(0,\sigma^{2}I_{n}\right)$ and $\varepsilon_{M,t}∼N_{k}\left(0,\sigma^{2}I_{k}\right)$. We then directly calculate the mutual information $I(x_{t+1},x_{t}|do\left(x_{t}\right)∼U\left({\left[-1,1\right]}^{n}\right))$ for micro-dynamics and $I(y_{t+1},y_{t}|do\left(y_{t}\right)∼U\left({\left[-1,1\right]}^{k}\right))$ between the generated samples of $x_{t+1}$ at time t+1 and the samples of $x_{t}$ at time *t* to obtain the numerical solution of effective information and calculate causal emergence as

(42) $$\begin{array}{c} \Delta I=\frac{I(y_{t+1},y_{t}|do\left(y_{t}\right)∼U\left({\left[-1,1\right]}^{k}\right))}{k}-\frac{I(x_{t+1},x_{t}|do\left(x_{t}\right)∼U\left({\left[-1,1\right]}^{n}\right))}{n}. \end{array}$$

To distinguish from analytical solutions ΔJ, we use ΔI to represent the causal emergence computed by the mentioned numeric method. From Figure 4e, we found that when n=4,k=1, and $\sigma^{2}=0.01$, as the sample size increases, the numerical solutions for causal emergence gradually approach the analytical solutions.

### 4.3. Spiral Rotating

In addition to the results obtained for specific models in Section 4.1 and Section 4.2, we need a more intuitive understanding of the meaning of coarse-graining and causal emergence. Here, we take a spiral rotating model [60] from analytical geometry [61,62] as an example to visualize our model system in $R^{3}$ space and analyze how causal emergence is reflected.

A common example is a model in which a rigid body rotates about an axis. Suppose we have a vector in three-dimensional space that represents the position of a point $x_{0}\in R^{3}$, and we want to rotate it around some straight line through the origin. This operation can be represented by the rotation matrix *R*. For rotation around the straight line represented by the unit vector u=(a,b,c), $\left||u|\right|=\sqrt{a^{2}+b^{2}+c^{2}}=1$, a rotation matrix can be used to describe the rotation operation:

(43) $$\begin{array}{c} \begin{array}{c} R=\left(\begin{array}{ccc} \cos\theta +a^{2}\left(1-\cos\theta \right) & ab(1-\cos\theta )-c\sin\theta & ac(1-\cos\theta )+b\sin\theta \\ ab(1-\cos\theta )+c\sin\theta & \cos\theta +b^{2}\left(1-\cos\theta \right) & bc(1-\cos\theta )-a\sin\theta \\ ac(1-\cos\theta )-b\sin\theta & bc(1-\cos\theta )+a\sin\theta & \cos\theta +c^{2}\left(1-\cos\theta \right) \end{array}\right), \end{array} \end{array}$$

where θ represents the angle of the rotation. This matrix describes a rotation about the straight line represented by the unit vector *u*. Multiply the vector $x_{t}$ by the matrix *R* to obtain the new rotated vector $x_{t+1}$. This rotation matrix describes a model of rotation around a certain straight line and can be used to describe rotation operations around a specified axis in three-dimensional space. Since *R* does not change the modulus of $x_{t}$, we can add an adjustment matrix $\Psi =\mathrm{diag}(\psi_{1},\psi_{2},\psi_{3})$ to adjust the modulus of the variable $x_{t}$. Then, the linear stochastic iteration systems in $R^{3}$ is

(44) $$\begin{array}{c} x_{t+1}=Ax_{t}+\varepsilon_{t},A=R\Psi ,\varepsilon_{t}∼N\left(0,\sigma^{2}I_{n}\right),\sigma =0.01, \end{array}$$

When $\psi_{i}<1,i=1,2,3$, the model is attenuated. Adjusting $\psi_{i}$ can cause the system to produce different values of causal emergence at different macro-state dimensions.

Assuming the direction vector of the rotation axis is $u_{0}={\left(0,0.1,1\right)}^{T}$, after normalization as $u=u_{0}/\left|\right|u_{0}\left|\right|$, the rotation axis *u* can be obtained. At the same time, we specify the rotation angle θ=π/16 and η=0 because the dimension averaged Shannon entropy of both $x_{t}$ and $y_{t}$ remains the same. Through θ and *u*, we can obtain the rotation matrix *R*. By changing Ψ, we will obtain causal emergence in different forms.

**Figure 5:**
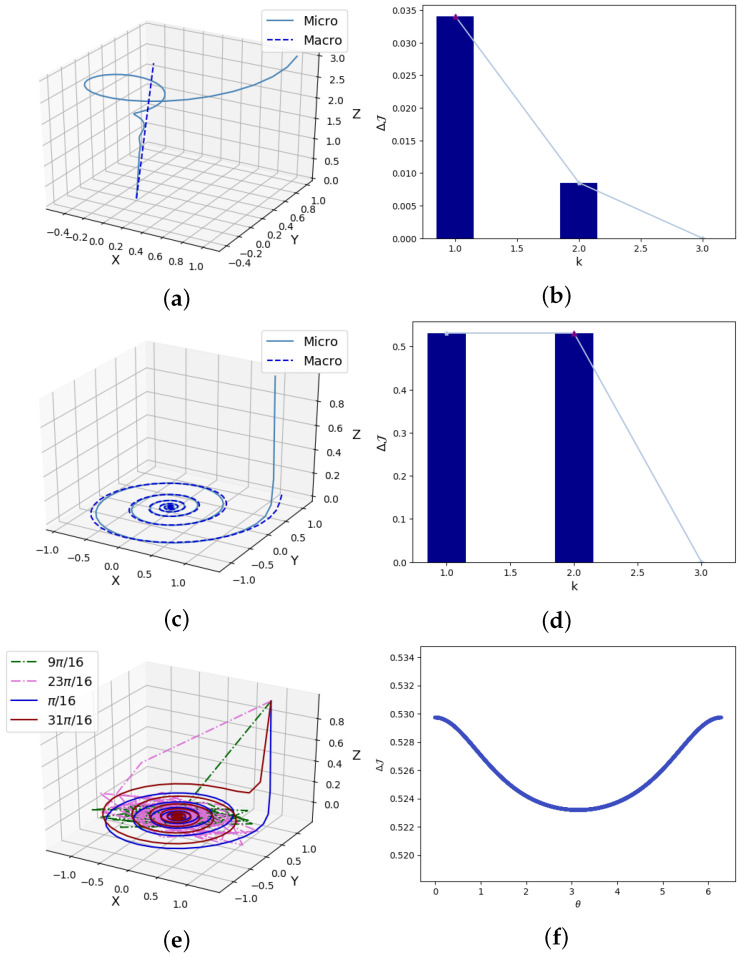
Experimental results of spiral rotating. (**a**) When Ψ=diag(0.94,0.94,0.99) and $x_{0}={\left(1,1,3\right)}^{T}$, $x_{t}$ represents a point in space that rotates around the axis of rotation and contracts towards the axis of rotation. By the optimal coarse-graining $W^{*}$, we can obtain macro-states that move along the axis of rotation. (**b**) The degree of causal emergence and its dependence on the dimension of macro-states for the example in (**a**), which reaches its maximum value as ΔJ=0.0341 when k=1. (**c**) The spiral curve when Ψ=diag(0.99,0.97,0.2) and $x_{0}={\left(1,1,1\right)}^{T}$; the trajectory of $x_{t}$ is compressed to a plane perpendicular to *u* at the initial stage. (**d**) We can project $x_{t}$ onto the plane as a macro-state, where k=2, ΔJ=0.5295. (**e**) When π/2<θ<3π/2, $x_{t}$ tends to oscillate more than rotate, while θ<π/2 or θ>3π/2$x_{t}$ tends to be a rotate model. (**f**) When θ approaches π, the degree of causal emergence is smaller, while when it approaches 0 or 2π, the degree of causal emergence is larger.

The occurrence of causal emergence in three-dimensional space can be understood as the stronger causal effect of system evolution when the path of $x_{t}$ is projected onto a plane or a straight line. As shown in Figure 5a, when Ψ=diag(0.94,0.94,0.99) and $x_{0}={\left(1,1,3\right)}^{T}$, $x_{t}$ contracts towards the axis of rotation. In Figure 5b, the causal emergence reaches its maximum value as ΔJ=0.0341 when k=1. The eigenvalues of the micro-state matrix *A* are $\lambda_{1}=0.9895$, $\lambda_{2}=0.9222+0.1834i$, and $\lambda_{3}=0.9222-0.1834i$; the macro-state parameter matrix $A_{M}$ only retains the eigenvalues with the maximum modulus $\lambda_{1}=0.9895$. This result is obtained by setting the coarse-graining mapping *W* to compress the rotation trajectory into a one-dimensional straight line as the macro-states.

In the second scenario in Figure 5c, when Ψ=diag(0.99,0.97,0.2) and $x_{0}={\left(1,1,1\right)}^{T}$, the path of $x_{t}$ will first shrink to a plane perpendicular to *u*. In Figure 5d, we project $x_{t}$ onto the plane as the macro-state, where k=2, ΔJ=0.5295. We retain the two eigenvalues $\lambda_{1}=0.9612+0.1900i$ and $\lambda_{2}=0.9612-0.1900i$ with the largest and identical module lengths. The reason why we do not take k=1 here is that the more dimensionality we reduce, the greater the information loss and $|\lambda_{1}\left|=\right|\lambda_{2}|$. Therefore, in the absence of further improvement in causal emergence, we do not need to continue the dimensionality reduction of the system.

As well as Ψ, rotation angle θ also significantly impacts causal emergence. In Figure 5f, when θ approaches π, the degree of causal emergence is smaller, while when it approaches 0 or 2π, it is larger. This is because, when π/2<θ<3π/2, $x_{t}$ tends to oscillate more than rotate, as shown in Figure 5e. The correlation between $x_{t}+1$ and $x_{t}$ in the model is relatively small, leading to a weakening of causal emergence.

## 5. Discussion

### 5.1. Optimal Causal Emergence When $W^{†}=W^{T}$

In previous sections, we have obtained the explicit expression for the maximum causal emergence and the optimal coarse-graining under the condition that the dimension averaged uncertainty loss by coarse-graining is bounded. However, under this condition, the competition between the non-degeneracy term, which is solely determined by *A*, and the determinism term, which is determined by *A* and Σ in the optimal causal emergence, cannot be observed, as shown in Equation (31).

Therefore, we will discuss the maximization of causal emergence under a different condition, which is

(45) $$W^{†}=W^{T}$$

in this section. Equation (45) means the coarse-graining map *W* can be decomposed as a projection by discarding the information in n−k dimensions and a rotation in a *k*-dimensional space without information loss. This also means the *k* first singular values are units, and the norm of *W* and Σ will not be excessively enlarged or reduced. In this case, as $W^{†}=W^{T}$, the coarse-graining strategy will simultaneously filter the eigenvalues of *A* and Σ. According to Equations (17) and (20), we can get that $\left|\det\left(A\right)\right|/\det{\left(\Sigma \right)}^{1/2}=\left|\det\left(A\Sigma^{-1/2}\right)\right|$. Therefore, $A\Sigma^{-1/2}$ determines the causal emergence of the system and it is necessary for it to be analyzed below.

**Theorem** **3.** *When $W^{†}=W^{T}$, the causal emergence of linear stochastic iterative systems satisfies*

(46) $$\begin{array}{c} \Delta J\leq \frac{1}{k}\sum_{i=1}^{k}\ln|{\tilde{d}}_{i}|-\frac{1}{n}\sum_{i=1}^{n}\ln\left|{\tilde{d}}_{i}\right|, \end{array}$$

*where $|{\tilde{d}}_{1}\left|\geq \cdots \geq \right|{\tilde{d}}_{n}|$ are n eigenvalues of $A\Sigma^{-1/2}$. The equal sign holds when A and Σ share the same n eigenvectors as*

(47) $$A\Sigma^{-1/2}=V\Lambda K^{-1/2}V^{T}=V\tilde{D}V^{T},$$

*where $\tilde{D}=\Lambda K^{-1/2}=\mathrm{diag}\left({\tilde{d}}_{1},\ldots ,{\tilde{d}}_{n}\right)$ is also a diagonal matrix, in which $|{\tilde{d}}_{1}\left|\geq \cdots \geq \right|{\tilde{d}}_{n}|$, and V is the matrix formed by the shared eigenvectors of A and Σ.*

**Figure 6:**
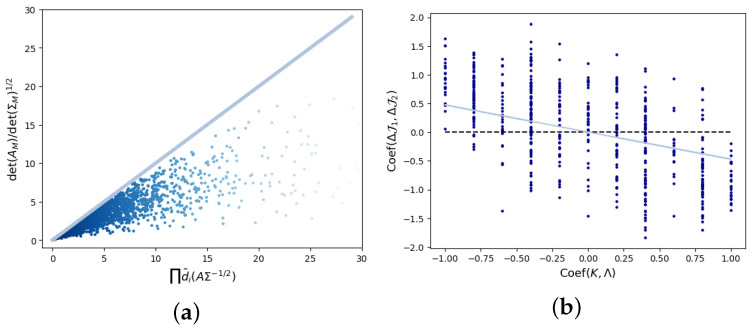
(**a**) We randomly generate matrices *A*, Σ, and *W* with a singular value of 1 when n=4, k=2; we can see that the upper bound of $|\det\left(WAW^{†}\right)|/\det{\left(W\Sigma W^{T}\right)}^{1/2}$ is ${\tilde{d}}_{1}{\tilde{d}}_{2}$. The steel blue straight line is $|\det\left(WAW^{†}\right)|/\det{\left(W\Sigma W^{T}\right)}^{1/2}={\tilde{d}}_{1}{\tilde{d}}_{2}$. (**b**) When $\lambda_{1}=0.8,\;\lambda_{1}=0.2,\;\lambda_{3}=0.4,\;\lambda_{4}=0.2$, and we randomly arrange (0.2,0.4,0.6,0.8) to generate $\left(\kappa_{1},\ldots ,\kappa_{n}\right)$. $C(\Delta J_{1},\Delta J_{2})$ and C(κ,λ) show a negative slope after drawing the scatter plot.

When *A* and Σ do not share the eigenvector matrix, we cannot directly calculate the correlation between the eigenvalue matrix of $A\Sigma^{-1/2}$ and *K* or Λ within the current framework of matrix theory, so it is difficult to find an analytical solution for causal emergence. However, we can still obtain numerical solutions through optimization of *W* and confirm that the upper bound of causal emergence is to retain the maximum *k* eigenvalues of $A\Sigma^{-1/2}$. We have the inequality (46) in Theorem 3. To verify this inequality, we randomly generate matrices *A*, Σ, and *W* with a singular value of 1 when n=4, k=2; we can see that the upper bound of $|\det\left(WAW^{†}\right)|/\det{\left(W\Sigma W^{T}\right)}^{1/2}$ is ${\tilde{d}}_{1}{\tilde{d}}_{2}$ in Figure 6b. Unlimited, the singular value of *W* means that it can reduce the macro-state noise and information entropy in the system. This also means that the locally effective information we retain can be adjusted arbitrarily by adjusting *W*. This is also a balance point between determinism and non-degeneracy.

Then, we can derive an analytical expression for the maximized causal emergence when *A* and Σ share the same *n* eigenvectors; that is to say, $\Sigma =VKV^{T}$ and $A=V\Lambda V^{T}$, and Equation (47) holds. Rearranging the order of $\tilde{d_{i}}$ can result in diagonal matrix $\tilde{D}=\mathrm{diag}\left({\tilde{d}}_{1},\ldots ,{\tilde{d}}_{n}\right)$, in which $|{\tilde{d}}_{1}\left|\geq \cdots \geq \right|{\tilde{d}}_{n}|$. Then, according to Equation (20), we can obtain an analytical solution for the maximized causal emergence, which is also the case where Equation (46) takes the equal sign. The above proof process can refer to Appendix B.

Next, we will study when the relationship between the determinism ($\Delta J_{2}$) and the non-degeneracy ($\Delta J_{1}$) is cooperative, such that the causal emergence can be maximized under the condition of the simultaneous diagonalization for *A* and Σ. This problem is equivalent to asking when the quantity expressed in Equation (46), which is determined by the eigenvalues of $A\Sigma^{-1/2}$, will be maximized if the eigenvalues in *A* and Σ are unchanged. We found that the orders of the eigenvalues in both *A* and Σ, which can be characterized by the correlation between $\kappa =(\kappa_{1},\ldots ,\kappa_{n})$ and $\lambda =(\lambda_{1},\ldots ,\lambda_{n})$, denoted as C(κ,λ), play an important role. If λ and κ are positively correlated, the effects of determinism and non-degeneracy contributed to the causal emergence cancel each other out. For example, when λ=(0.8,0.6,0.4,0.2), κ=(0.8,0.6,0.4,0.2), and k=2, the optimal coarse-graining strategy for $\Delta J_{1}$ should retain the largest k=2 eigenvalues $\lambda_{1}=0.8,\lambda_{2}=0.6$ of *A* according to Equation (28), but for κ, the largest *k* eigenvalues $\kappa_{1}=0.8,\kappa_{2}=0.6$ are also retained, which implies small determinism and large non-degeneracy. We can see that the determinism and the non-degeneracy are mutually inhibiting, leading to a small causal emergence: ΔJ=0.

On the other hand, if λ and κ are negatively correlated, then causal emergence can be increased because determinism and non-degeneracy reinforce each other. For example, when λ=(0.8,0.6,0.4,0.2) and κ=(0.2,0.4,0.6,0.8), the optimal coarse-graining strategy for $\Delta J_{1}$ should retain the largest k=2 eigenvalues $\lambda_{1}=0.8,\lambda_{2}=0.6$ of *A*; this also means retaining the smallest *k* eigenvalues of Σ, then determinism and non-degeneracy are mutually reinforced, and a larger causal emergence can be obtained: ΔJ=0.8959.

In Figure 6a, we visualize how the correlation between λ and κ can influence the cooperative relationship between determinism and non-degeneracy, which can be characterized by the correlation between $\Delta J_{1}$ and $\Delta J_{2}$, denoted as $C(\Delta J_{1},\Delta J_{2})$ in a small example. First, we fix the values and the orders of Λ as $\lambda_{1}=0.8,\;\lambda_{1}=0.2,\;\lambda_{3}=0.4,$ and $\lambda_{4}=0.2$. Second, we randomly permute (0.2,0.4,0.6,0.8) to generate κ. Finally, we randomly sample the invertible matrix *V* such that *A* and Σ can be obtained. The results are shown in Figure 6a; $C(\Delta J_{1},\Delta J_{2})$ and C(κ,λ) show a negative slope after drawing the scatter plot. This means that the two values are negatively correlated. In general, maximizing causal emergence ΔJ is also a process of finding optimal orders to arrange $\lambda_{i}$ and $\kappa_{i}$ for all *i* to achieve a balance between determinism $\Delta J_{2}$ and non-degeneracy $\Delta J_{1}$.

### 5.2. About the Information Loss in Dynamics Prediction

Our entire paper discusses the changes in the causal emergence of systems after coarse-graining. For linear stochastic iteration systems, deleting smaller eigenvalues in the parameter matrix *A* or larger eigenvalues in Σ can both increase the value of causal emergence. Coarse graining and data dimensionality reduction [37,38,39,40,41] are intricately linked. Once data dimensionality is reduced, it will be accompanied by information loss, and the transformation from micro-states to macro-states is no exception. Therefore, we also need to briefly analyze the relationship between the loss function in dynamics prediction and the effective information of the dynamics. For linear stochastic iteration systems, we define the dynamical loss in the following way:

**Definition** **6.** *(Dynamical loss): For the variable $x_{t}$ at time t, we define the dynamical loss as*

(48) $$\begin{array}{c} L_{D}=\left|\right|x_{t+1}-{\tilde{x}}_{t+1}\left|\right|, \end{array}$$

*in which $x_{t+1}=Ax_{t}+\varepsilon_{t}$, ${\tilde{x}}_{t+1}=\phi^{†}\left(y_{t+1}\right)=W^{†}y_{t+1}$.*

In Definition 6, $y_{t+1}$ is derived from $y_{t}$ and the macro iterative Equation (8) and $y_{t}=Wx_{t}$. Therefore,

(49) $$\begin{array}{c} {\tilde{x}}_{t+1}=W^{†}WAW^{†}Wx_{t}+W^{†}W\varepsilon_{t}, \end{array}$$

the dynamical loss then should be:

(50) $$\begin{array}{c} L_{D}=\left|\right|\left(W^{†}WAW^{†}W-A\right)x_{t}+\left(W^{†}W-I\right)\varepsilon_{t}\left|\right|. \end{array}$$

This is the difference between the micro-state variables $x_{t}\in R^{n}$ that are then mapped into micro-states after one iteration in the macro dynamics and the actual micro-state $x_{t+1}$, which reflects the information loss caused by coarse-graining on dynamics.

Due to the dependence between $L_{D}$ and variables $x_{t}$ and $\varepsilon_{t}$, in order to directly analyze the relationship between the causal emergence of the system ΔJ and the dynamical loss, we consider the upper bound of $L_{D}$ to eliminate the influence of random variables.

**Lemma** **3.** (Supremum of dynamical loss): For the variable $x_{t}$ at time t, when $\left|\right|x_{t}{\left|\right|}^{*}=\sup_{t}\left|\right|x_{t}\left|\right|<\infty$, we define the supremum of dynamical loss $L_{D}$ as

(51) $$\begin{array}{c} L_{D}\leq S_{D}=\underset{Non-degeneracySupremum}{||A-\hat{A}{\left|\right|}_{F}\left|\right|x_{t}{\left|\right|}^{*}}+\underset{DeterminismSupremum}{\left(n-k\right)||\varepsilon_{t}\left|\right|}, \end{array}$$

*in which $\hat{A}=W^{†}WAW^{†}W$. $S_{D}$ can be decomposed into determinism supremum and non-degeneracy supremum. $\left|\right|\cdot {\left|\right|}_{F}$ is the Frobenius norm of matrix ·.*

We can directly determine the relationship between the upper bound and $\Delta J_{1}$. When *k* is a constant, the minimum value of $S_{D}$ is equivalent to the maximum value of non-degeneracy emergence, $\Delta J_{1}$.

**Theorem** **4.** *($\Delta J_{1}$ and $S_{D}$): For the known variables $x_{t}$, the micro-state parameter matrix A and the noise $\varepsilon_{t}$, t=0,1,...,*

(52) $$\begin{array}{c} \arg\max_{W}\Delta J_{1}=\arg\min_{W}S_{D}=W^{*}. \end{array}$$

The proof of this theorem is referred to in Appendix C. After obtaining the relationship between the supremum $S_{D}$ and the non-degeneracy emergence $\Delta J_{1}$, it can be observed that after minimizing $S_{D}$, the solution set of *W*, $W_{L}$, satisfies $W_{L}\in W^{*}$. It can be seen that reducing loss can only increase the non-degeneracy of the system, but a further search for *W* is needed for determinism. This explains why we still need to train for a few steps to find the maximum causal emergence after the loss function converges in machine learning for causal emergence identification [35,43].

Through this indicator, we can easily see that there are two situations that can ensure that the system is under control. One is that the model is a decay model, that is,

(53) $$\begin{array}{c} |\lambda_{n}\left|<\cdots <\right|\lambda_{1}|<1, \end{array}$$

where $\lambda_{i}$ are eigenvalues of matrix *A*. The cases in Section 4.2 and Section 4.3 all meet this condition, ensuring the validity of the analysis.

The second possible situation is that we specify the time range of evolution,

(54) $$\begin{array}{c} \exists \tau <\infty ,s.t.\forall t>\tau ,x_{t}=0. \end{array}$$

In this way, even if the system is divergent and will grow infinitely over time, it will become 0 after time τ, making $x_{t}<\infty$. We used this method in Section 4.1 to make our evolution controllable within the specified time 0≤t≤100. Although the future cannot be measured, as shown by the black dashed line in Figure 3a, we can confirm that the data is bounded within the specified time range.

### 5.3. Nonlinear Form

Although the effective information and causal emergence in this paper are based on linear stochastic iterative systems, we can still apply them to nonlinear models under certain conditions. Nonlinear stochastic iterative systems like $x_{t+1}=f\left(x_{t}\right)+\varepsilon_{t}$ do not have the same known parameter matrix *A* as linear stochastic iterative systems. However, when $f:R^{n}\to R^{n}$, we can replace *A* with the gradient matrix of $\nabla f\left(x_{t}\right)\in R^{n\times n}$ at $x_{t}$.

Therefore, near $x_{t}$, when the noise $\varepsilon_{t}$ and the length of intervention space *L* are small, we can obtain an expression for the effective information of the nonlinear iterative system,

(55) $$\begin{array}{c} \begin{array}{cc} J(f\left(x_{t}\right),\Sigma ) & =\ln\left(\frac{L}{{\left(2\pi e\right)}^{1/2}}\right)+\frac{1}{L^{n}}\int_{{\left[-\frac{L}{2},\frac{L}{2}\right]}^{n}}\ln{\left|\frac{\det\left(\nabla f(x_{t})\right)}{\det{\left(\Sigma \right)}^{\frac{1}{2}}}\right|}^{\frac{1}{n}}dx_{t}\\ = & \ln\frac{|\det\left(\nabla f\left(x_{t}^{\prime }\right)\right){|}^{\frac{1}{n}}L}{{\left(2\pi e\right)}^{\frac{1}{2}}\det{\left(\Sigma \right)}^{\frac{1}{2n}}}\approx \ln\frac{|\det\left(\nabla f\left(x_{t}\right)\right){|}^{\frac{1}{n}}L}{{\left(2\pi e\right)}^{\frac{1}{2}}\det{\left(\Sigma \right)}^{\frac{1}{2n}}} \end{array}, \end{array}$$

as $x_{t}\approx x_{t}^{\prime }\in {\left[-L/2,L/2\right]}^{n}$; $x_{t}^{\prime }$ is the differential mean value in the Lagrange mean value theorem.

Analogous to linear iterative systems, we can infer the macro-state expression of nonlinear iterative systems,

(56) $$\begin{array}{c} f_{M}\left(y_{t}\right)=\phi \left(f\left(\phi^{†}\left(y_{t}\right)\right)\right), \end{array}$$

as $y_{t}=\phi \left(x_{t}\right)$ and $\phi :R^{n}\to R^{k},k<n$. $\phi^{†}:R^{k}\to R^{n},\phi \left(\phi^{†}\left(y_{t}\right)\right)=y_{t}$.

In this way, we can obtain the expression of causal emergence as

(57) $$\begin{array}{c} \Delta J=\ln\frac{|\det(\nabla f_{M}\left(y_{t}\right){|}^{\frac{1}{k}}}{|\det(\nabla f\left(x_{t}\right){|}^{\frac{1}{n}}}+\ln\frac{{\left|\det\left(\Sigma \right)\right|}^{\frac{1}{2n}}}{|\det\left(\Sigma_{M}\right){|}^{\frac{1}{2k}}}. \end{array}$$

For some simple differentiable nonlinear functions $f(x_{t})$, we can use this method to calculate the magnitude of causal emergence.

## 6. Conclusions

In this article, we first propose the coarse-graining strategy $y_{t}=Wx_{t}$ of linear stochastic iterative systems like $x_{t+1}=Ax_{t}+\varepsilon_{t},\varepsilon_{t}∼N\left(0,\Sigma \right)$, which map micro-states $x_{t}\in R^{n}$ into macro-states $y_{t}\in R^{k},k<n$. Coarse-graining can naturally derive macro dynamical models $y_{t+1}=A_{M}y_{t}+\varepsilon_{M,t}$ for $y_{t}\in R^{k}$, $A_{M}=WAW^{†}$, $\varepsilon_{M,t}∼N\left(0,\Sigma_{M}\right)$, $\Sigma_{M}=W\Sigma W^{T}$. For $x_{t+1}=Ax_{t}+\varepsilon_{t}$, we can use J and ΔJ to represent the effective information of the system and causal emergence in different dimensions *k* and *n*. To ensure that the macro dynamical information entropy $H(p\left(y_{t+1}|y_{t}\right))$ is not excessively reduced during coarse-graining, we need to ensure that $\frac{H(p\left(x_{t+1}|x_{t}\right))}{n}-\frac{H(p\left(y_{t+1}|y_{t}\right))}{k}$ is bounded by η when $do\left(y_{t}\right)∼U\left({\left[-L/2,L/2\right]}^{k}\right)$ and $do\left(x_{t}\right)∼U\left({\left[-L/2,L/2\right]}^{n}\right)$.

To directly determine the causal emergence in the system without being affected by coarse-graining parameter matrix *W*, we optimize *W* to obtain the maximum causal emergence $\Delta J^{*}$ that the system can achieve. When obtaining the optimal solution, the iterative parameter matrix $A_{M}$ only retains the maximum *k* eigenvalues of *A*, and the determinant value of $\Sigma_{M}$ is compressed to the lowest allowed lower limit $\epsilon \det{\left(\Sigma \right)}^{\frac{k}{n}}$. The obtained solution set can be understood as the intersection of *k* hyperplanes and hyperellipsoids, an ellipse in three-dimensional space. We search for causal emergence in three systems that are known models, focusing on the application of determinism, non-degeneracy, and the manifestation of causal emergence in $R^{3}$ space. In random walks, energy dissipation, and accurate selection models, varying degrees of causal emergence can be detected.

We conclude that there are two influencing factors for causal emergence; one is the iterative parameter matrix *A* and the other is the covariance matrix Σ of random noise, which respectively affect the non-degeneracy and determinism of the system. Smaller eigenvalues in the parameter matrix indicate weak causal relationships between variables in certain dimensions. Suppose the coarse-grained strategy precisely discards these dimensions. In that case, the causal effect of the macro-state dynamics is stronger than that of the micro-state, and the optimal causal emergence will occur.

The causal emergence calculation method in this article has several advantages. Firstly, we present the causal emergence of a known model in a linear stochastic iterative system as an analytical solution. This is also the first time that causal emergence has been calculated as an analytical solution in a continuous-state dynamic system. Secondly, in maximizing causal emergence, we found the solution set of the coarse-graining strategy. We found that the eigenvalues of the parameter matrix in a linear system determine the value of causal emergence. The third is the final result we obtain, which is only related to the system itself and not to specific data. This, to some extent, solves the problem of numerical solutions relying on data and the need for artificial search for coarse-graining strategies.

However, our model still has many shortcomings. One is that the current model is only applicable to linear systems, and there is still a lack of unified analysis and judgment for nonlinear systems. On the other hand, the time of the linear stochastic iterative system we study is discrete, and the calculation method of causal emergence is not applicable to continuous time dynamic systems. Combing EI with relevant work about the dynamic interaction between neural spike trains [63] could be a possible direction to solve the problem.

There are three important research directions in the future. One is to consider how to correctly calculate causal emergence for continuous-time Markov processes with discrete probability space, such as birth and death processes or queuing processes. The other direction concerns the causal emergence of stochastic differential equations or Fokker–Plank equations [64,65], such as option pricing equations and Langevin equations. The third direction is to quantify and explore the causal relationship between EI and unknown models, such as Granger causality [52] or the causal effect of neural spike trains [63]. We will continue to conduct in-depth research on these contents in the future.

## Data Availability

All the codes and data are available at: https://github.com/kilovoltage/An_Exact_Causal_Emergence_Theory (accessed on 21 May 2024).

## Appendix A. Calculation Effective Information and Causal Emergence

In this appendix, we will prove the theorems related to effective information and causal emergence and demonstrate the specific process of formula derivation. The first process to be demonstrated is the inference of effective information related to linear stochastic iterative systems J.

**Proof** **(Calculation process of Definition 4).** For the linear stochastic iteration system like Equation (1), $x_{t+1}$ follows a normal distribution about $x_{t}$, so $x_{t+1}∼N\left(Ax_{t},\Sigma \right)$,

(A1) $$\begin{array}{c} p\left(x_{t+1}|x_{t}\right)=\frac{1}{\det{\left(\Sigma \right)}^{\frac{1}{2}}{\left(2\pi \right)}^{\frac{n}{2}}}\exp\left\{-\frac{1}{2}{\left(x_{t+1}-Ax_{t}\right)}^{\prime }\Sigma^{-1}\left(x_{t+1}-Ax_{t}\right)\right\} \end{array}$$

Following the calculation method of causal geometry, assuming $\varepsilon_{t}$ is small, $do\left(x_{t}∼U\left({\left[-L/2,L/2\right]}^{n}\right)\right)\equiv do\left(x_{t}∼U\right)$, then $p\left(x_{t}\right)=1/L^{n},L>0$, we can calculate the effect distribution.

(A2) $$\begin{array}{c} \begin{array}{cc} E_{D}\left(x_{t+1}\right) & =\int_{U}p\left(x_{t+1}|x_{t}\right)p\left(x_{t}\right)d^{n}x_{t}\\ & =\int_{U}\frac{1}{\det{\left(\Sigma \right)}^{\frac{1}{2}}{\left(2\pi \right)}^{\frac{n}{2}}}\exp\left\{-\frac{1}{2}{\left(x_{t+1}-Ax_{t}\right)}^{\prime }\Sigma^{-1}\left(x_{t+1}-Ax_{t}\right)\right\}\frac{1}{L^{n}}d^{n}x_{t}\\ {\;}_{\left(x_{t}\approx A^{-1}x_{t+1}\right)} & \approx \int_{X}\frac{1}{\det{\left(\Sigma \right)}^{\frac{1}{2}}{\left(2\pi \right)}^{\frac{n}{2}}}\exp\left\{-\frac{1}{2}{\left(x_{t+1}-Ax_{t}\right)}^{\prime }\Sigma^{-1}\left(x_{t+1}-Ax_{t}\right)\right\}\frac{\left|\det(A^{-1})\right|}{L^{n}}d^{n}x_{t+1}\\ & =\frac{1}{\left|\det\left(A\right)\right|L^{n}} \end{array} \end{array}$$

Afterward, as $X={\left[-\infty ,\infty \right]}^{n}$ is domain of $x_{t+1}$, we can calculate the effective information as

(A3) $$\begin{array}{c} \begin{array}{cc} EI & =\int_{X}D_{KL}\left[p\left(x_{t+1}|x_{t}\right)|E_{D}\left(x_{t+1}\right)\right]d^{n}x_{t+1}\\ & =\int_{U}\frac{d^{n}x_{t}}{L^{n}}\int_{X}\frac{1}{\det{\left(\Sigma \right)}^{\frac{1}{2}}{\left(2\pi \right)}^{\frac{n}{2}}}\exp\left\{-\frac{1}{2}{\left(x_{t+1}-Ax_{t}\right)}^{\prime }\Sigma^{-1}\left(x_{t+1}-Ax_{t}\right)\right\}\\ & \left[-\ln\left(\det{\left(\Sigma \right)}^{\frac{1}{2}}{\left(2\pi \right)}^{\frac{n}{2}}\right)-\frac{1}{2}{\left(x_{t+1}-Ax_{t}\right)}^{\prime }\Sigma^{-1}\left(x_{t+1}-Ax_{t}\right)-\ln\frac{1}{\left|\det\left(A\right)\right|L^{n}}\right]d^{n}x_{t+1}\\ & =-\frac{1}{2}\ln\left(\det\left(\Sigma \right){\left(2\pi \right)}^{n}\right)-\frac{n}{2}-\ln\frac{1}{\left|\det\left(A\right)\right|L^{n}}\\ & =\ln\frac{\left|\det\left(A\right)\right|L^{n}}{\det{\left(\Sigma \right)}^{\frac{1}{2}}{\left(2\pi e\right)}^{\frac{n}{2}}}. \end{array} \end{array}$$

According to the properties of information entropy, we use determinism information,

(A4) $$\begin{array}{c} \begin{array}{cc} -{\langle H(p\left(x_{t+1}|x_{t}\right))\rangle }_{x_{t}} & =-{\langle -\int_{X}p\left(x_{t+1}|x_{t}\right)\ln\left(p\left(x_{t+1}|x_{t}\right)\right)d^{n}x_{t+1}\rangle }_{x_{t}}\\ & =\int_{U}\frac{d^{n}x_{t}}{L^{n}}\int_{X}\frac{1}{\det{\left(\Sigma \right)}^{\frac{1}{2}}{\left(2\pi \right)}^{\frac{n}{2}}}\exp\left\{-\frac{1}{2}{\left(x_{t+1}-Ax_{t}\right)}^{\prime }\Sigma^{-1}\left(x_{t+1}-Ax_{t}\right)\right\}\\ & \left[-\ln\left(\det{\left(\Sigma \right)}^{\frac{1}{2}}{\left(2\pi \right)}^{\frac{n}{2}}\right)-\frac{1}{2}{\left(x_{t+1}-Ax_{t}\right)}^{\prime }\Sigma^{-1}\left(x_{t+1}-Ax_{t}\right)\right]d^{n}x_{t+1}\\ & =\left[-\ln\left(\det{\left(\Sigma \right)}^{\frac{1}{2}}{\left(2\pi \right)}^{\frac{n}{2}}\right)-\frac{n}{2}\right]\\ & =\ln\frac{1}{\det{\left(\Sigma \right)}^{\frac{1}{2}}{\left(2\pi e\right)}^{\frac{n}{2}}} \end{array}, \end{array}$$

and non-degeneracy information,

(A5) $$\begin{array}{c} \begin{array}{cc} H(E_{D}\left(x_{t+1}\right)) & =-\int_{X}E_{D}\left(x_{t+1}\right)\ln\left(E_{D}\left(x_{t+1}\right)\right)d^{n}x_{t+1}\\ {\;}_{\left(x_{t}\approx A^{-1}x_{t+1}\right)} & \approx -\int_{X}E_{D}\left(x_{t+1}\right)\ln\frac{1}{\left|det\left(A\right)\right|L^{n}}d^{n}x_{t+1}\\ & =\ln\left(\left|det\left(A\right)\right|L^{n}\right) \end{array}. \end{array}$$

It is obvious that

(A6) $$\begin{array}{c} -{\langle H(p\left(x_{t+1}|x_{t}\right))\rangle }_{x_{t}}+H\left(E_{D}\left(x_{t+1}\right)\right)=\ln\frac{\left|\det\left(A\right)\right|L^{n}}{\det{\left(\Sigma \right)}^{\frac{1}{2}}{\left(2\pi e\right)}^{\frac{n}{2}}}=EI. \end{array}$$

Then, we can easily get

(A7) $$\begin{array}{c} J\equiv \frac{EI}{n}=\frac{1}{n}\ln\frac{\left|\det\left(A\right)\right|L^{n}}{{\left(2\pi e\right)}^{\frac{n}{2}}\det{\left(\Sigma \right)}^{\frac{1}{2}}}=\ln\frac{{\left|\det\left(A\right)\right|}^{\frac{1}{n}}L}{{\left(2\pi e\right)}^{\frac{1}{2}}\det{\left(\Sigma \right)}^{\frac{1}{2n}}}. \end{array}$$

If the system is nonlinear, it can be understood that $A=\nabla f(x_{t})$ will change with $x_{t}$. Then, Equations (55) and (57) can also be proven. □

After obtaining effective information, we can calculate causal emergence.

**Proof** **of** **Theorem** **1.** For the micro dynamical systems like Equation (1) and macro dynamical systems like Equation (8) after coarse-graining, we can calculate the micro effective information $J_{m}$ and macro effective information $J_{M}$ of two kinds of dynamical systems separately. In this way, causal emergence is

(A8) $$\begin{array}{c} \begin{array}{cc} \Delta J & =J_{M}-J_{m}=\frac{EI_{M}}{k}-\frac{EI_{m}}{n}\\ & =\frac{1}{k}\ln\frac{|\det\left(A_{M}\right)|L^{k}}{\det{\left(\Sigma_{M}\right)}^{\frac{1}{2}}{\left(2\pi e\right)}^{\frac{k}{2}}}-\frac{1}{n}\ln\frac{\left|\det\left(A\right)\right|L^{n}}{\det{\left(\Sigma \right)}^{\frac{1}{2}}{\left(2\pi e\right)}^{\frac{n}{2}}}\\ & =\frac{1}{k}\underset{DeterminismInformation\;Non-degeneracyInformation}{\left(\ln\frac{1}{\det{\left(\Sigma_{M}\right)}^{\frac{1}{2}}{\left(2\pi e\right)}^{\frac{k}{2}}}+\ln\left(|\det\left(A_{M}\right)|L^{k}\right)\right)}\\ & -\frac{1}{n}\left(\ln\frac{1}{\det{\left(\Sigma \right)}^{\frac{1}{2}}{\left(2\pi e\right)}^{\frac{n}{2}}}+\ln\left(\left|\det\left(A\right)\right|L^{n}\right)\right)\\ & =\underset{Non-degeneracyEmergence}{\ln\frac{|\det\left(WAW^{†}\right){|}^{\frac{1}{k}}}{{\left|\det\left(A\right)\right|}^{\frac{1}{n}}}}+\underset{DeterminismEmergence}{\ln\frac{{\left|\det\left(\Sigma \right)\right|}^{\frac{1}{2n}}}{|\det\left(W\Sigma W^{T}\right){|}^{\frac{1}{2k}}}}. \end{array} \end{array}$$

□

## Appendix B. Optimization of Macrodynamic Parameter Matrix

To maximize causal emergence, we need to use relevant content from matrix theory to optimize coarse-graining strategies.

**Proof** **of** **Lemma** **1.** Perform eigendecomposition on matrix *A*, $A=V\Lambda V^{-1}$, $\Lambda =\mathrm{diag}(\lambda_{1},$$\ldots ,\lambda_{n})\in R^{n\times n}$ is the diagonal matrix of eigenvalues, $V\in R^{n\times n}$ is the eigenvector matrix corresponding to the eigenvalue matrix. Determinant

(A9) $$\begin{array}{c} \det\left(WAW^{†}\right)=\det\left(WV\Lambda V^{-1}W^{†}\right)=\det\left(\tilde{W}\Lambda {\tilde{W}}^{†}\right)=\sum_{m}\left(\gamma_{m}\prod_{\lambda_{i}\in L_{m}}\lambda_{i}\right), \end{array}$$

in which $L_{m}=\left\{\lambda_{N_{m,1}},\lambda_{N_{m,2}},\ldots ,\lambda_{N_{m,k}}\right\}\subseteq \left\{\lambda_{1},\ldots ,\lambda_{n}\right\}$ are new sets formed by arbitrarily choosing *k* eigenvalues from *n* eigenvalues of *A*. The sorting of these sets is based on the sum of the elements in the set, in descending order. $N_{m}\subseteq \left\{1,\ldots ,n\right\}$, the number of elements in sets $|N_{m}\left|,\right|L_{m}|=k$, $m=1,2,\ldots ,C_{n}^{k}$. $\gamma_{m}$ are coefficients determined by *W*, $\gamma_{m}>0$, $\sum_{m}\gamma_{m}=1$. It is not difficult to find that when $\gamma_{m}=1$, matrix *A* only retains the eigenvalues in $L_{m}$ after transformation. The calculation of $\gamma_{1},\ldots ,\gamma_{C_{n}^{k}}$ is as follows. Set $WV=\tilde{W}={\left(w_{ij}\right)}_{k\times n}$, then $W^{†}V^{-1}={\tilde{W}}^{†}={\left(v_{ij}\right)}_{n\times k}$. According to the principle of determinant calculation, we can divide $\gamma_{m}$ into two parts,

(A10) $$\begin{array}{c} \gamma_{m}=\gamma_{m,1}-\gamma_{m,2}, \end{array}$$

where $\gamma_{m,1},\gamma_{m,2}$ correspond to the sum of terms with coefficients of 1 and −1 in the determinant calculation, respectively. Therefore,

(A11) $$\begin{array}{c} \gamma_{m,1}=\sum_{l=0}^{k-1}\left[\sum_{s\in {\left\{s\right\}}_{m}}\left(\prod_{i=1}^{k}w_{i,s_{i}}v_{s_{i},j}\right)\right],j=\left\{\begin{array}{cc} i+l,i+l & \leq k\\ i+l-k,i+l & >k \end{array}\right., \end{array}$$

(A12) $$\begin{array}{c} \gamma_{m,2}=\sum_{l=2}^{k+1}\left[\sum_{s\in {\left\{s\right\}}_{m}}\left(\prod_{i=1}^{k}w_{i,s_{i}}v_{s_{i},j}\right)\right],j=\left\{\begin{array}{cc} l-i,l-i & >0\\ l-i+k,l-i & \geq 0 \end{array}\right.. \end{array}$$

where $s=\{s_{1},s_{2},...s_{k}\}$ is a new sequence formed by randomly arranging all elements in $N_{m}$; ${\left\{s\right\}}_{m}$ is a set composed of such elements *s*. Number of elements in the set ${|\left\{s\right\}}_{m}|=k!$. If all eigenvalues of *A* satisfy $\lambda_{1}\geq \lambda_{2}\geq \cdots \geq \lambda_{n}$, we can simultaneously find the maximum and minimum values of $\det(WAW^{†})$. When $\gamma_{1}=1$, only the maximum *k* eigenvalues are retained, and $\det(WAW^{†})$ takes the maximum value $\prod_{i=1}^{k}\lambda_{i}$. On the contrary, when $\gamma_{m}=1$, only the smallest *k* eigenvalues are retained, and the $\det(WAW^{†})$ takes the minimum value $\prod_{i=n-k+1}^{n}\lambda_{i}$. Therefore,

(A13) $$\begin{array}{c} \prod_{i=n-k+1}^{n}\lambda_{i}\leq \det\left(WAW^{†}\right)\leq \prod_{i=1}^{k}\lambda_{i}. \end{array}$$

From the proof of (A13), we know that $\gamma_{m}$ are coefficients determined by *W* and when $\gamma_{m}=1$, matrix *A* only retains the eigenvalues in $L_{m}$. Therefore, when the eigenvalues of matrix *A* are not specified to be all positive real numbers, the modulus length satisfies $|\lambda_{1}\left|\geq \right|\lambda_{2}\left|\geq \cdots \geq \right|\lambda_{n}|$, then

(A14) $$\begin{array}{c} \begin{array}{c} |\det\left(WAW^{†}\right)|=\left|\sum_{m}\left(\gamma_{m}\prod_{\lambda_{i}\in L_{m}}\lambda_{i}\right)\right|\leq \sum_{m}\gamma_{m}\left|\prod_{\lambda_{i}\in L_{m}}\lambda_{i}\right|=\sum_{m}\gamma_{m}\left(\prod_{\lambda_{i}\in L_{m}}\left|\lambda_{i}\right|\right)\leq \prod_{i=1}^{k}\left|\lambda_{i}\right|. \end{array} \end{array}$$

We directly study the situation of eigenvalues in the complex field. When $L_{m}=\lambda_{1},\ldots ,\lambda_{k}$, the determinant $\left|\det(WAW^{†})\right|$ reaches its maximum value. The decrease of $\gamma_{m}$ and the intervention of smaller absolute values of eigenvalues $|\lambda_{i}|,i\geq k$ lead to the decrease of $\left|\det(WAW^{†})\right|$. □

**Proof** **of** **Theorem** **3.** According to (A9) and Jensen’s inequality, we can obtain inequalities

(A15) $$\begin{array}{c} \begin{array}{c} |\det\left(W\Sigma W^{†}\right){|}^{-\frac{1}{2}}={\left[\sum \left(\gamma_{m}\prod \kappa_{i}\right)\right]}^{-\frac{1}{2}}\leq \left[\sum \gamma_{m}{\left(\prod \kappa_{i}\right)}^{-\frac{1}{2}}\right]=\left|\det\left(W\Sigma^{-\frac{1}{2}}W^{†}\right)\right|, \end{array} \end{array}$$

if and only if the eigenvalues are equal; the equal sign holds. By utilizing this inequality and combining it with the relevant properties of linear algebra, we can conclude that

(A16) $$\begin{array}{c} \begin{array}{cc} \Delta J & =\ln\frac{|\det\left(WAW^{†}\right){|}^{\frac{1}{k}}}{|\det\left(W\Sigma W^{†}\right){|}^{\frac{1}{2k}}}-\ln\frac{{\left|\det\left(A\right)\right|}^{\frac{1}{n}}}{{\left|\det\left(\Sigma \right)\right|}^{\frac{1}{2n}}}\\ & =\frac{1}{k}\ln(|\det\left(W\Sigma W^{†}\right){|}^{-\frac{1}{2}}|\det\left(WAW^{†}\right)\left|\right)-\frac{1}{n}\ln\left|\Sigma^{-\frac{1}{2}}A\right|\\ & \leq \frac{1}{k}\ln(|\det\left(W\Sigma^{-\frac{1}{2}}W^{†}\right)||\det\left(WAW^{†}\right)\left|\right)-\frac{1}{n}\ln\left|\Sigma^{-\frac{1}{2}}A\right|\\ & =\frac{1}{k}\ln|\det\left(W\Sigma^{-\frac{1}{2}}W^{†}WAW^{†}\right)|-\frac{1}{n}\ln\left|\Sigma^{-\frac{1}{2}}A\right|\\ {\;}_{\left(\Sigma =VKV^{T},A=V\Lambda V^{T}\right)} & \leq \frac{1}{k}\ln|\det\left(W\Sigma^{-\frac{1}{2}}AW^{†}\right)|-\frac{1}{n}\ln\left|\Sigma^{-\frac{1}{2}}A\right|\\ {\;}_{\left(W=\left({\tilde{W}}_{k},O_{k\times \left(n-k\right)}\right)V^{T}\right)} & \leq \frac{1}{k}\sum_{i=1}^{k}\ln|{\tilde{d}}_{i}|-\frac{1}{n}\sum_{i=1}^{n}\ln\left|{\tilde{d}}_{i}\right| \end{array} \end{array}$$

When *A* and Σ share the same *n* eigenvectors, that is to say, $\Sigma =VKV^{T}$ and $A=V\Lambda V^{T}$, where *V* is the matrix formed by the shared eigenvectors and *K* and Λ are the eigenvalues for *A* and Σ, respectively, the eigenvalues of $\Sigma^{-1/2}A$ are equal to the product of the eigenvalues of *A* and $\Sigma^{-1/2}$. $W=\left({\tilde{W}}_{k},O_{k\times \left(n-k\right)}\right)V^{T}$ represents that both *A* and $\Sigma^{-1/2}$ retain the eigenvalues with the highest norm; the second and third unequal signs can also be taken as equal signs. □

## Appendix C. Loss Function and Causal Emergence

In this section, we first prove that $S_{D}$ is the supremum of $L_{D}$.

**Proof** **of** **Lemma** **3.** Based on the theory of matrix modules, when $\left|\right|x_{t}{\left|\right|}^{*}=\sup_{t}\left|\right|x_{t}\left|\right|<\infty$,

(A17) $$\begin{array}{c} \begin{array}{cc} L_{D} & =||Ax_{t}+\varepsilon_{t}-\left(W^{†}WAW^{†}Wx_{t}+W^{†}W\varepsilon_{t}\right)\left|\right|\\ & =\left|\right|\left(A-W^{†}WAW^{†}W\right)x_{t}+\left(I_{n}-W^{†}W\right)\varepsilon_{t}\left|\right|\\ & \leq \left|\right|\left(A-W^{†}WAW^{†}W\right)x_{t}\left|\right|+\left|\right|\left(I_{n}-W^{†}W\right)\varepsilon_{t}\left|\right|\\ & \leq \left|\right|\left(A-W^{†}WAW^{†}W\right){\left|\right|}_{F}\left|\right|x_{t}\left|\right|+\left|\right|\left(I_{n}-W^{†}W\right){\left|\right|}_{F}\left|\right|\varepsilon_{t}\left|\right|\\ & =\left|\right|\left(A-W^{†}WAW^{†}W\right){\left|\right|}_{F}\left|\right|x_{t}\left|\right|+\left(n-k\right)\left|\right|\varepsilon_{t}\left|\right|\\ & \leq \left|\right|\left(A-W^{†}WAW^{†}W\right){\left|\right|}_{F}\left|\right|x_{t}{\left|\right|}^{*}+\left(n-k\right)\left|\right|\varepsilon_{t}\left|\right|=S_{D} \end{array}. \end{array}$$

□

Next, we prove that *W* at the minimum value of the supremum $S_{D}$ can maximize the non-degeneracy emergence.

**Proof** **of** **Lemma** **4.** $|\lambda_{1}\left|\geq \right|\lambda_{2}\left|\geq \cdots \geq \right|\lambda_{n}|\geq 0$ are *n* modulus of eigenvalues of matrix *A* sorted from largest to smallest. $\Lambda =\mathrm{diag}\left(\lambda_{1},\ldots ,\lambda_{n}\right)\in R^{n\times n}$. $\tilde{W}=WV$, ${\tilde{W}}^{†}\tilde{W}$ and $I-{\tilde{W}}^{†}\tilde{W}$ are both symmetric idempotent matrices. $rk({\tilde{W}}^{†}\tilde{W})=k$, $I-{\tilde{W}}^{†}\tilde{W}={\left(r_{ij}\right)}_{n\times n}$ has the following properties:

(1) $\sum_{l=1}^{n}r_{il}r_{lj}=r_{ij}$.
(2) $||I-{\tilde{W}}^{†}\tilde{W}{\left|\right|}_{F}=\sqrt{\sum_{i,j=1}^{n,n}r_{ij}^{2}}=k$.
(3) $\forall l,0<l\leq n,\sqrt{\sum_{i,j=1}^{n,n}{\left(r_{il}r_{lj}\right)}^{2}}=1$

Based on the theory of matrix modules,

(A18) $$\begin{array}{c} \begin{array}{cc} S_{D} & =\left|\right|\left(A-W^{†}WAW^{†}W\right){\left|\right|}_{F}\left|\right|x_{t}\left|\right|+\left(n-k\right)\left|\right|\varepsilon_{t}\left|\right|\\ {\;}_{\left(\tilde{W}=WV\right)} & =||V\left(\Lambda -{\tilde{W}}^{†}\tilde{W}\Lambda {\tilde{W}}^{†}\tilde{W}\right)V^{-1}{\left|\right|}_{F}\left|\right|x_{t}\left|\right|+\left(n-k\right)\left|\right|\varepsilon_{t}\left|\right|\\ & =||\Lambda -{\tilde{W}}^{†}\tilde{W}\Lambda {\tilde{W}}^{†}\tilde{W}{\left|\right|}_{F}\left|\right|x_{t}\left|\right|+\left(n-k\right)||\varepsilon_{t}\left|\right|\\ & =\left|\right|\left(I_{n}-{\tilde{W}}^{†}\tilde{W}\right)\Lambda \left(I_{n}-{\tilde{W}}^{†}\tilde{W}\right)+2\left(I_{n}-{\tilde{W}}^{†}\tilde{W}\right)\Lambda {\tilde{W}}^{†}\tilde{W}{\left|\right|}_{F}\left|\right|x_{t}\left|\right|+\left(n-k\right)\left|\right|\varepsilon_{t}\left|\right|\\ & \geq \left|\right|\left(I_{n}-{\tilde{W}}^{†}\tilde{W}\right)\Lambda \left(I_{n}-{\tilde{W}}^{†}\tilde{W}\right){\left|\right|}_{F}\left|\right|x_{t}\left|\right|+\left(n-k\right)\left|\right|\varepsilon_{t}\left|\right|\\ & =\sqrt{\sum_{0<i,j\leq n}{\left|\sum_{l=1}^{n}r_{il}r_{lj}\lambda_{l}\right|}^{2}}\left|\right|x_{t}\left|\right|+\left(n-k\right)||\varepsilon_{t}\left|\right|\\ & \geq \sqrt{\sum_{0<i,j\leq n}\sum_{l=1}^{n}{\left|r_{il}r_{lj}\lambda_{l}\right|}^{2}}\left|\right|x_{t}\left|\right|+\left(n-k\right)||\varepsilon_{t}\left|\right|\\ & \geq \sqrt{\sum_{0<i,j\leq n}\sum_{l=k+1}^{n}{\left|r_{il}r_{lj}\lambda_{l}\right|}^{2}}\left|\right|x_{t}\left|\right|+\left(n-k\right)||\varepsilon_{t}\left|\right|\\ & \geq \sqrt{\sum_{l=k+1}^{n}{\left|\lambda_{l}\right|}^{2}}\left|\right|x_{t}\left|\right|+\left(n-k\right)||\varepsilon_{t}\left|\right| \end{array}, \end{array}$$

if and only if the maximum *k* eigenvalues are preserved in $\hat{A}$, only the n−k eigenvalues with the smallest modulus are retained, $S_{D}$; the equal sign holds. $W=\left({\tilde{W}}_{k},O_{k\times \left(n-k\right)}\right)V^{-1}$ is exactly the same solution set as the non-degeneracy emergence at its maximum $\Delta J_{1}^{*}$. □
